# Population-level single-cell genomics reveals conserved gene programs in systemic juvenile idiopathic arthritis

**DOI:** 10.1172/JCI166741

**Published:** 2023-11-15

**Authors:** Emely L. Verweyen, Kairavee Thakkar, Sanjeev Dhakal, Elizabeth Baker, Kashish Chetal, Daniel Schnell, Scott Canna, Alexei A. Grom, Nathan Salomonis, Grant S. Schulert

**Affiliations:** 1 Division of Rheumatology and; 2 Division of Biomedical Informatics, Cincinnati Children’s Hospital Medical Center, Cincinnati, Ohio, USA.; 3 Department of Pharmacology and Systems Physiology, University of Cincinnati College of Medicine, Cincinnati, Ohio, USA.; 4 Children’s Hospital of Philadelphia, Division of Rheumatology, Philadelphia, Pennsylvania, USA.; 5 Department of Pediatrics, University of Cincinnati College of Medicine, Cincinnati, Ohio, USA.

**Keywords:** Inflammation, Arthritis, Lupus, Monocytes

## Abstract

Systemic autoimmune and autoinflammatory diseases are characterized by genetic and cellular heterogeneity. While current single-cell genomics methods provide insights into known disease subtypes, these analysis methods do not readily reveal novel cell-type perturbation programs shared among distinct patient subsets. Here, we performed single-cell RNA-Seq of PBMCs of patients with systemic juvenile idiopathic arthritis (SJIA) with diverse clinical manifestations, including macrophage activation syndrome (MAS) and lung disease (LD). We introduced two new computational frameworks called UDON and SATAY-UDON, which define patient subtypes based on their underlying disrupted cellular programs as well as associated biomarkers or clinical features. Among twelve independently identified subtypes, this analysis uncovered what we believe to be a novel complement and interferon activation program identified in SJIA-LD monocytes. Extending these analyses to adult and pediatric lupus patients found new but also shared disease programs with SJIA, including interferon and complement activation. Finally, supervised comparison of these programs in a compiled single-cell pan-immune atlas of over 1,000 healthy donors found a handful of normal healthy donors with evidence of early inflammatory activation in subsets of monocytes and platelets, nominating possible biomarkers for early disease detection. Thus, integrative pan-immune single-cell analysis resolved what we believe to be new conserved gene programs underlying inflammatory disease pathogenesis and associated complications.

## Graphical abstract

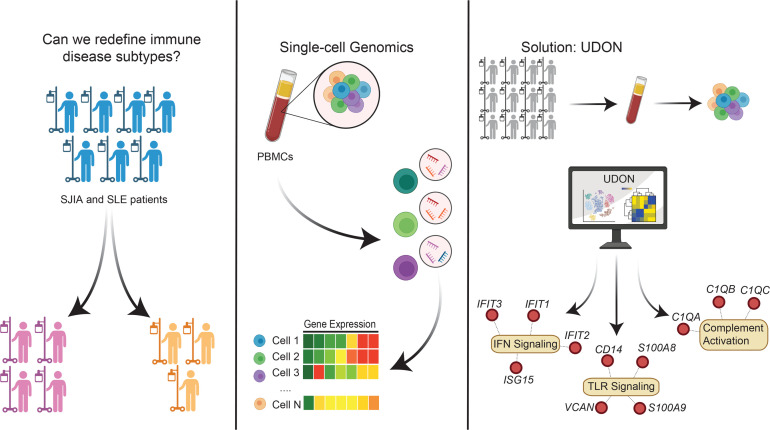

## Introduction

Diseases of the immune system associated with systemic autoimmunity and autoinflammation can result in significant health burdens, which, in many disorders, include a high risk for life-threatening complications. Inflammatory diseases are believed to evolve from complex underlying etiologies, including both genetic and environmental, and include common disorders such as type I diabetes to relatively rare but serious disorders such as systemic juvenile idiopathic arthritis (SJIA). The immunopathogenesis of SJIA is multifaceted, with features of myeloid activation, autoimmunity, classical autoinflammation, and interferonopathy linked to disease heterogeneity and complications (1). Recently, single-cell RNA-Seq (scRNA-Seq) has elucidated underlying gene expression and genetic associations, through independent population-level and focused diseased cohort analyses in disorders such as systemic lupus erythematosus (SLE) (2). Such analyses have led to important insights into the impact of gene expression in myeloid and lymphoid cell populations that are associated with common genetic variation across nearly a thousand healthy donors (3).

While single-cell genomics provides the opportunity to understand cellular, molecular, and genetic associations among patients and people in the control group, existing approaches are focused on harmonization of cells across patients without considering novel patient subsets associated with distinct gene regulatory programs (4). Such analyses are crucial in systemic inflammatory disorders such as SJIA and SLE, in which patients are characterized by diverse disease states associated with different inflammatory signaling and immune cell–specific impacts, that often remain largely unknown. Indeed, given the large incidence of autoimmune disease within the population (approximately 7%), it is likely that many presumably healthy patients have underlying autoimmune dysfunction that has not yet manifested in diagnosed disease. Hence, new integrative analysis and strategies that leverage large cohorts of control and patient samples from distinct inflammatory disorders are required to begin understanding the novel common and unique autoimmune cellular programs and their functional relationship to patient phenotypes.

To address these challenges, we developed a computational framework designed to exploit differences in the gene expression programs of individual patients versus people who are presumed to be healthy controls, at the level of individual cell type. This workflow, unsupervised discovery of novel disease programs, or UDON, is designed to discover both known and new disease subtypes associated with coherent gene-expression differences unique to a subset of patients. These subtypes comprise both patient samples and cell types associated with distinct gene expression modules. By discerning such patient/cell type subsets, we can readily identify clinical (e.g., histologic) or laboratory (e.g., biomarker) covariates for each cell type that are associated with different UDON clusters, we call this Statistical Association Test for ClinicAl PhenotYpes (SATAY-UDON). First, to assess the ability of this approach to resolve new disease subsets, we performed what is, to our knowledge, the first in-depth scRNA-Seq of a comprehensive cohort of patients with SJIA with clinically diverse etiologies (active disease, inactive disease, SJIA-associated lung disease [SJIA-LD], SJIA-macrophage activation syndrome [SJIA-MAS]) and matched pediatric healthy controls. In addition to resolving entirely new cellular and molecular subtypes of SJIA, these analyses identify peripheral biomarkers that predict distinct inflammatory responses, including multiple divergent myeloid phenotypes. We found a complement and IFN activation program enriched in SJIA-LD monocytes and confirmed from the serum of independent patients. To broadly assess systemic inflammatory disease subtypes, we performed a comprehensive pan-immune single-cell survey of de novo subtypes in over 1,000 individuals with SLE and healthy donors, to identify both SJIA-specific as well as broadly conserved transcriptional programs. These data provide critical insights into SJIA pathogenesis, the broader transcriptional landscape present in systemic inflammatory diseases, and the conserved inflammatory programs in subsets of healthy donors.

## Results 

### PBMC populations defined in children with SJIA, SJIA-MAS, and SJIA-LD.

**Figure 1:**
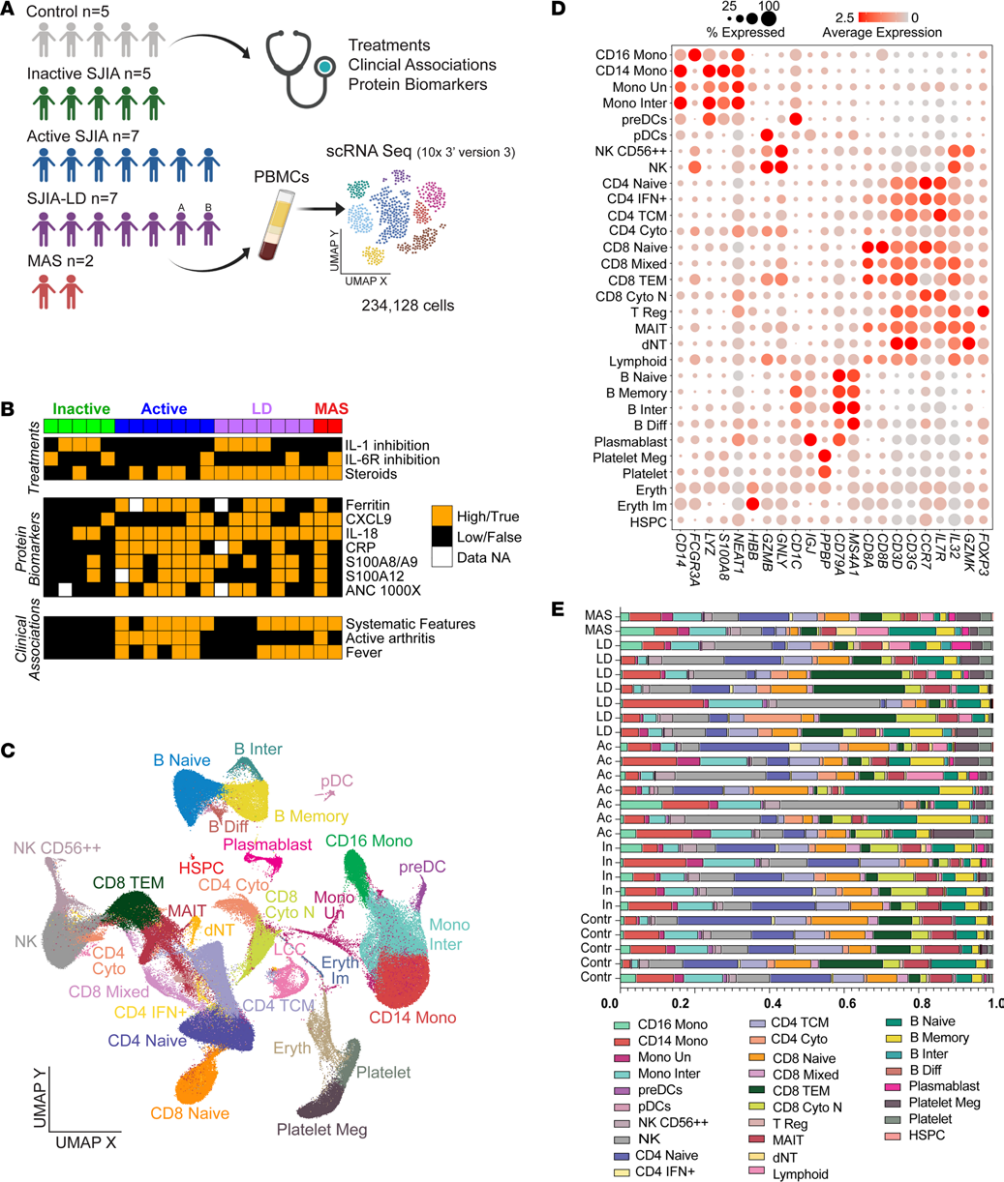
PBMCs vary in composition by pediatric SJIA clinical subtype. (**A**) Study overview illustrating 26 patients with SJIA and people in the control group, for which clinical features were collected and PBMCs were analyzed by scRNA-Seq. (**B**) Binary plot depicting treatments, laboratory parameters (protein biomarkers), and systemic features (clinical associations) of each patient at time of sample collection. (**C**) Integrated UMAP of 234,128 single cells and 30 annotated cell populations from SJIA samples and controls. Cluster identity specified on the basis of Azimuth and literature associations. (**D**) Dot plot of average population gene expression for prior-defined cell type marker genes. Dot size indicates the percentage of cells expressing the gene and color intensity indicates the mean expression. (**E**) Bar plot indicating cell frequency of each cell type (erythrocytes excluded) per sample in the cohort shown in panel **A**. In, inactive SJIA; Ac, active SJIA; LD, SJIA-LD; MAS, SJIA-MAS.

While adult and pediatric forms of SLE have been extensively characterized through scRNA-Seq (2), other severe systemic inflammatory disorders, such as SJIA, have not. To define the immune landscape of SJIA, we performed scRNA-Seq analysis on PBMCs from a cohort of patients across disease activity and complications. Clinical disease course and treatment response in SJIA is highly variable; in addition, some children experience potentially fatal episodes of SJIA-MAS. SJIA-MAS represents a systemic cytokine storm syndrome considered a form of secondary hemophagocytic lymphohistiocytosis (HLH) characterized by decreased cytolytic function, excessive activation of hemophagocytic macrophages, and expansion of T cells (5, 6). SJIA-LD is an increasingly recognized pulmonary complication encompassing varying levels of pulmonary alveolar proteinosis (PAP), pulmonary artery hypertension, and fibrosis. 80% of patients with SJIA-LD also have a history of MAS, suggesting that these complications are pathogenically linked (7, 8). Samples were obtained from 20 children with SJIA (5 inactive SJIA, 7 active SJIA, 7 SJIA-LD (6 individual patients, 1 patient sampled twice indicated as “A” and “B”), and 2 SJIA-MAS), as well as 5 individuals in the pediatric healthy control group (Figure 1A). All patients with active SJIA and SJIA-MAS, and 4 of 7 patients with SJIA-LD, had clinical features of active disease at time of sampling. Treatments included biologic therapy and/or steroids for most patients; some patients with active SJIA were newly diagnosed and sampled before initiation of therapy (Figure 1B and Supplemental Table 1; supplemental material available online with this article; https://doi.org/10.1172/JCI166741DS1). Laboratory parameters including serum ferritin, IL-18, CXCL9, and S100 proteins were frequently elevated particularly in samples from patients with active SJIA, SJIA-MAS, and SJIA-LD, while most parameters were normal in patients with inactive SJIA (Supplemental Table 2 and Figure 1B).

We integrated all patient and healthy donor PBMCs (*n* = 234,128 cells), considering possible donor and disease differences, to produce a compendium of 30 candidate cell populations (Figure 1C, Methods). These populations were annotated using a well-curated PBMC reference data set using Azimuth (9) and manual annotation (Methods, Supplemental Tables 3 and 4). These included all major blood constituents, including B cells, T cells, monocytes, dendritic cells (DCs), natural killer (NK) cells, erythrocytes, and platelets, in addition to sub-cell types with distinct marker genes (Figure 1D). No donor-specific cell populations were observed, except certain erythrocyte populations were highly enriched for a single patient with MAS, which is a common observation in patients with highly active SJIA-MAS and HLH (Supplemental Figure 1) (10).

Comparing cell-type frequencies among patients and controls in this integrated compendium nominates somewhat consistent differences among these clinically defined patient subsets (Figure 1E and Supplemental Figure 1). We find that patients with active SJIA and SJIA-LD have, by trend, increased NK cells, while platelets and platelet megakaryocytes were lesser in controls and patients with inactive SJIA. Additionally, there was a significantly lower proportion of mucosal-associated invariant T (MAIT) cells in all patients compared with people in the control group, while erythrocytes and double negative T cells (dNTs) increased by trend in the SJIA-MAS patients (Figure 1E and Supplemental Figures 1 and 2). Significantly lower proportions (over 5%, *t* test *P* value under 0.05) of CD4 naive and CD8 TEM cells were observed in patients with SJIA-LD as well as active and inactive SJIA, respectively, compared with people in the control group (Supplemental Table 3).

### SJIA-MAS patients are distinguished from other SJIA patients by a highly expressed IFN gene signature.

**Figure 2:**
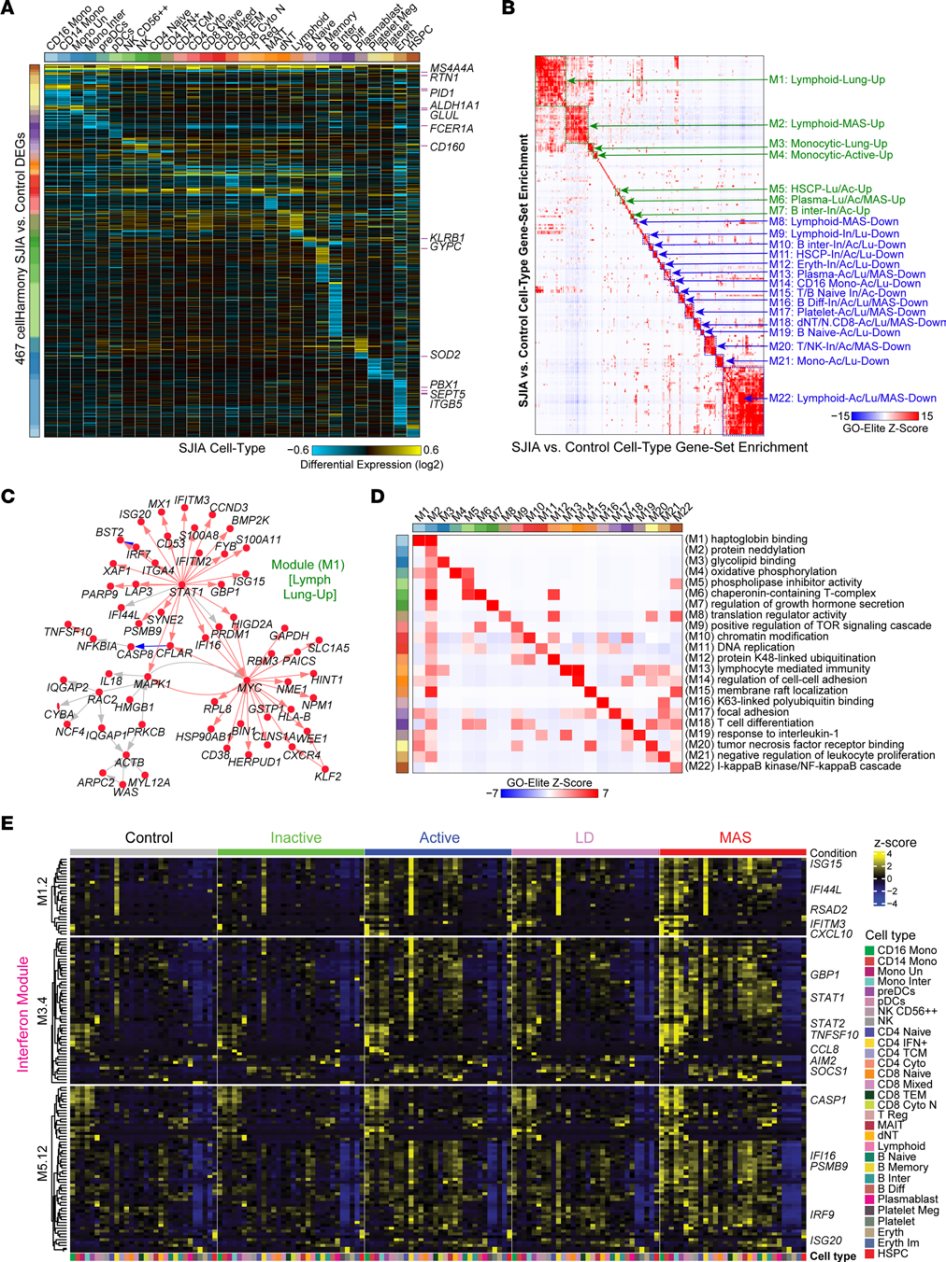
Patients with SJIA-MAS are distinguished from other patients with SJIA by a distinct IFN gene signature. (**A**) cellHarmony differential fold–change heatmap comparing SJIA patient cell pseudo-bulks in disease (Active, LD, MAS) versus controls. Each column is the mean fold difference for a cell population and each row a gene (fold > 1.2 and empirical Bayes moderated *t* test *P* < 0.005, unadjusted). (**B**) Identification of SJIA impacted gene sets (modules), defined from all constituent cellHarmony SJIA subtype and cell-type comparisons. Each module represents multiple up or downregulated patient versus control signatures with mutual gene-set enrichments (GO-Elite). The source signatures include aggregate disease and specific SJIA subtypes versus controls. Module annotations (right) denote the major associated cell-types and subtypes signatures present. (**C**) Transcription factor (TF) and gene interaction networks for shared genes in module M1 from panel **B**. Red nodes indicate upregulation and Blue nodes indicate downregulation. Red arrows indicate annotated TF-target interactions in GO-Elite (TRRUST, Pazar, Amadeus). (**D**) Module-specific example Gene Ontology terms associated with each of the shared genes for each Module in panel **B**. (**E**) Heatmap of scaled (z-score) expression values of IFN-induced gene modules M1.2, M3.4 and M5.12 as described in Banchereau et al, 2016 (19), across all PBMC cell clusters and clinical groups.

Distinctive, but often overlapping, transcriptional signatures have been identified in SJIA, including IL-1, IL-18, Toll-like receptors (TLRs), and inflammasome signaling (11–14), while SJIA complications MAS and LD have been linked to both type I and type II IFN-pathway activation (7, 15, 16). In order to define dysregulated gene expression signatures in SJIA, we first identified differentially expressed genes (DEGs) comparing all patients for each clinical subtype against all people in the control group using the software cellHarmony (17). Here, rather than compare individual cells, we pooled all cells from each patient cell population into cell-type pseudobulks for disease versus healthy control comparisons, to increase rigor. cellHarmony found that the transcriptional landscape of patient cells from SJIA-LD and SJIA-MAS samples was more dysregulated than for patients with clinically inactive or active SJIA. SJIA-MAS MAIT cells were the most dysregulated cell-type with 614 upregulated DEGs (Supplemental Figure 3). Comparing all patients with active SJIA, SJIA-MAS, and SJIA-LD into a combined disease group against only the people in the healthy control group, we observed 467 commonly deregulated genes, including 18 genes associated with myeloid populations and previously described as significantly up or downregulated in a PBMCs bulk gene expression study (13) (Figure 2A).

We assessed cellHarmony-derived signatures using gene set enrichment to identify the unique and shared gene programs among these different clinical subsets. Pairwise comparison of all 306 SJIA-regulated cellHarmony gene sets (up and downregulated), identified 22 overlapping gene clusters of modules (M) shared in at least 3 signatures, typically associated with different SJIA subtypes in the same or related cell-types (Figure 2B, Methods). The largest module represented shared downregulated genes in diverse lymphoid cell populations, principally associated with SJIA-LD but including SJIA-MAS and active SJIA (M22). The other 2 largest modules represent upregulated genes shared among lymphoid cell populations in SJIA-LD (M1) or SJIA-MAS (M2), which showed weak but significant association with each other. Other commonly dysregulated cell-type gene programs were found in Monocytic (M3, M4, M14, M21), Plasmablast (M6, M13), B cell (M7, M10, M16, M19), T/NK cell (M15, M18, M20), platelets (M17), erythroid (M12), and HSCP (M5, M11). Gene-network analysis of these sets highlighted important commonalities and differences. Specifically, *STAT1* and *MYC* were predicted as key transcriptional regulators in SJIA-LD lymphoid populations (M1), while *STAT1* and *IRF1* were the dominant predicted regulators in SJIA-MAS populations (M2), suggesting both commonalities and differences in the IFN signature in these patient types (Figure 2C and Supplemental Figure 4). While much smaller, the downregulated lymphoid (M16) and B cell differentiation (M22) modules were also denoted by different predicted core regulators (*CTCF* and *RBL2* versus *JUN* and *JUND*, respectively) (Supplemental Figure 4). Comparison of dysregulated Gene Ontology terms showed M2 (SJIA-MAS) resulted in the broadest group of transcripts corresponding to diverse processes, while the other sets tended to show more specific modulation of processes involved in epigenetic regulation, focal adhesion, proliferation, and inflammatory, TNF, and mTOR signaling, among others (Figure 2D). Hence, these data indicate both shared and unique gene networks that differentially impinge upon broad chromatin regulators and inflammatory signaling pathways.

The above findings and previous studies suggest that IFNs play a key role in the disease pathogenesis of SJIA-MAS and SJIA-LD. To further determine IFN responses across PBMC, we performed visualization of previously defined IFN modules from whole blood, which finds substantial variation in the cellular source for different IFN-mediated genes (Figure 2E) (2, 7, 16, 18, 19). Specifically, we observe a shared CD4 IFN^+^-cell specific induction of IFN genes in active SJIA, SJIA-LD, and SJIA-MAS associated with a subset of IFN-targets (most pronounced in M1.2, which is predominantly driven by IFNβ, a type I IFN) and expanding pan-IFN response most strongly in SJIA-MAS, spanning the majority of myeloid, lymphoid, and B cell populations (particularly in the IFNγ driven M3.4 and M5.12) (Figure 2E and Supplemental Figure 5). Such impacts were not observed in people in the control group or patients with inactive SJIA, which had few upregulated IFN related genes.

### Transcriptional activation in monocytes and changes in lymphocyte cell frequency separate ongoing disease from inactive SJIA and controls.

**Figure 3:**
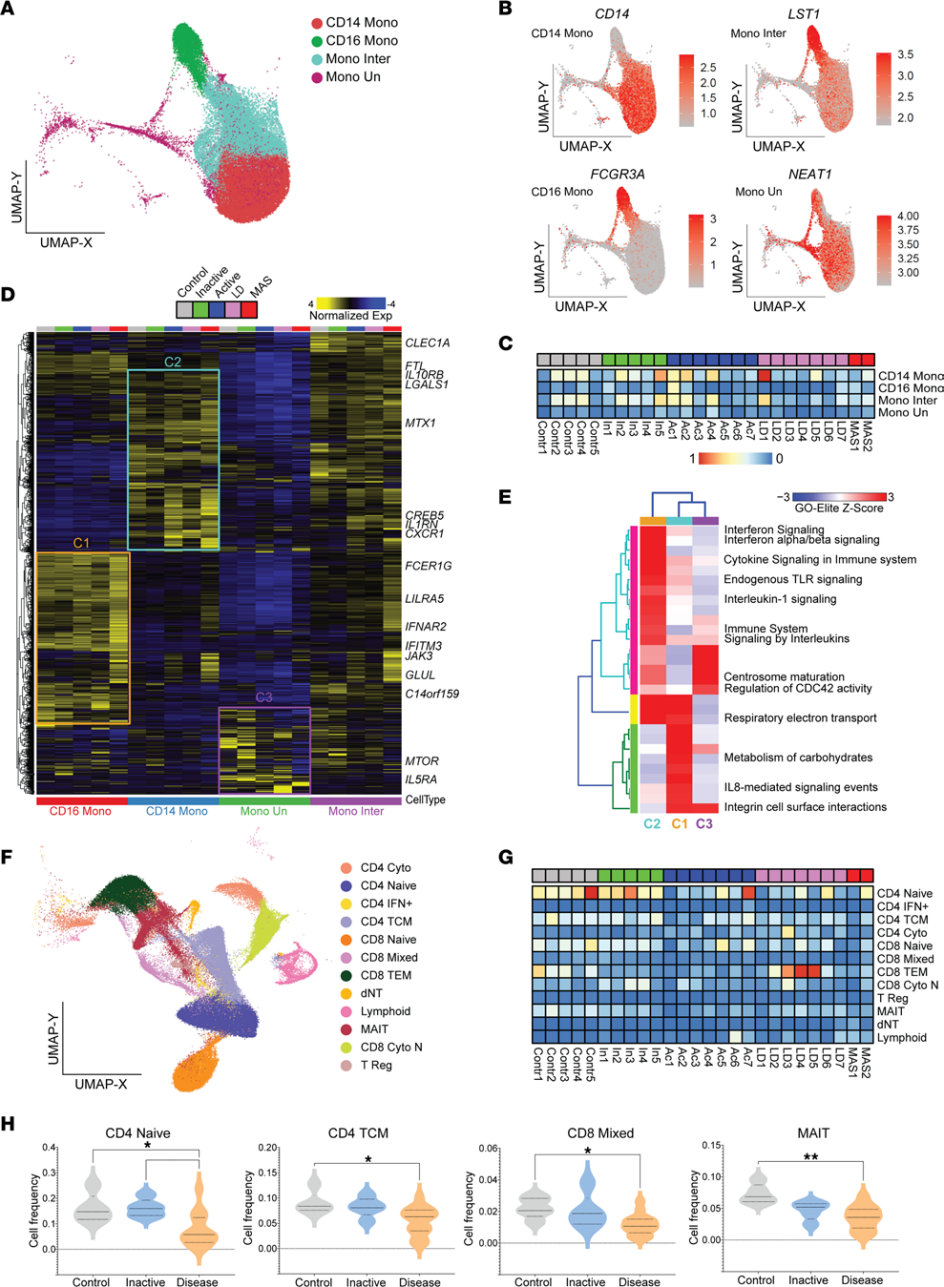
Transcriptional activation in monocytes and changes in lymphocyte cell frequency separate ongoing disease from inactive SJIA and controls. (**A**) UMAP representing all 4 monocytic cell populations identified by scRNA-Seq. (**B**) Feature plots indicating the expression levels of selected marker genes of monocyte populations. (**C**) Matrix representing the cell frequency per individual SJIA patient or control for the 4 monocytic cell populations. (**D**) Gene expression heatmap of previously determined monocyte signatures of high ferritin SJIA patients as described in Schulert et al, 2020 (20), across the 4 monocytic populations. Supervised clustering defined clusters C1, C2, and C3 shown in **D**, and GO-Elite analysis of associated cluster pathways (Pathway Commons) is shown in **E**. (**F**) UMAP representing all 12 T cell populations identified by scRNA-Seq. (**G**) Matrix representing cell frequency per individual SJIA patient or control. (**H**) Violin plots depicting significant differences in cell frequency in the 4 T cell populations between controls (*n* = 5), inactive SJIA (*n* = 5) or disease (combined data from active SJIA, SJIA-LD and SJIA-MAS) (*n* = 16). Bars indicate significant differences calculated by 1-way ANOVA (*P_adj_ ≤ 0.05, ***P_adj_ ≤ 0.005).

Monocytes are considered as central pathogenic drivers of SJIA and targeting of monocyte-derived proinflammatory cytokines is considered first line therapy. We identified 4 distinct monocytic populations in our patient compendium, CD14^+^ (classical), CD16^+^ (nonclassical), intermediate, and an unclassified population (monocyte undefined) (Figure 3, A and B and Supplemental Tables 3 and 4). These populations had varying frequency among individual patients, although 6 of 7 SJIA-LD samples showed fewer intermediate monocytes (Figure 3C). These intermediate monocytes presented a mix of CD14 and CD16 monocyte features, with fewer discriminating features. We questioned how previously described alternations in SJIA monocytes mapped across these cellular subpopulations. Supervised comparison to a prior blood monocyte gene signature from patients with SJIA with high serum ferritin (20) found 3 distinct gene modules, segregated by these myeloid populations (Figure 3D). Cluster 1 genes were enriched in IL-8 signaling markers, with the highest expression in CD16^+^ monocytes in SJIA-MAS (Figure 3E). Cluster 2 was characterized by preferential expression in CD14^+^ monocytes in active disease groups (Figure 3D), with enrichment for IL-1 signaling, endogenous TLR signaling, and IFN signaling (Figure 3E). Cluster 3 was most dominant in the undefined monocyte population, enriched in centrosome and mitosis genes and included *MTOR*, *IL5RA*, and *IL11RA* (Figure 3, D and E). Hence, we find monocytic subsets that are preferentially enriched, not restricted to distinct SJIA clinical phenotypes, and associated with multiple signaling and proliferative processes.

We next examined the relative abundance and activation state of lymphocyte populations in SJIA. Here, we found profound differences in the distribution of T cell populations identified across patients with SJIA (Figure 3F and Supplemental Figure 6). Considering all disease groups (active SJIA, SJIA-LD, and SJIA-MAS) relative to inactive SJIA and the control group, we found a broad decrease in CD4 naive, CD4 TCM, CD8 mixed cells, and MAIT cells (Figure 3, G and H). Recent data suggest the cytokine environment in SJIA may alter T cell polarization and represent a new possible avenue of therapy (21). Analysis of T cell polarization marker genes for Th1, Th2, and Th17 showed slightly higher expression of both Th1 (*CCL4, IFNG,* and *TBX21*) and Th2 (*CCR3* and *CCR6*) markers in active SJIA, SJIA-LD, and MAS (Supplemental Figure 7), suggesting a shift from more naive to more active T cell populations in SJIA. While there is some patient-level variation, we observed no significant differences in relative abundance of the other main cell-types (B cells, NK cells, Platelets and Erythrocytes, DCs, and HSCPs) (Supplemental Figures 8–11).

### Unsupervised discovery with UDON finds complement activation in the monocytes of patients with SJIA-LD and a subset of patients with active SJIA.

**Figure 4:**
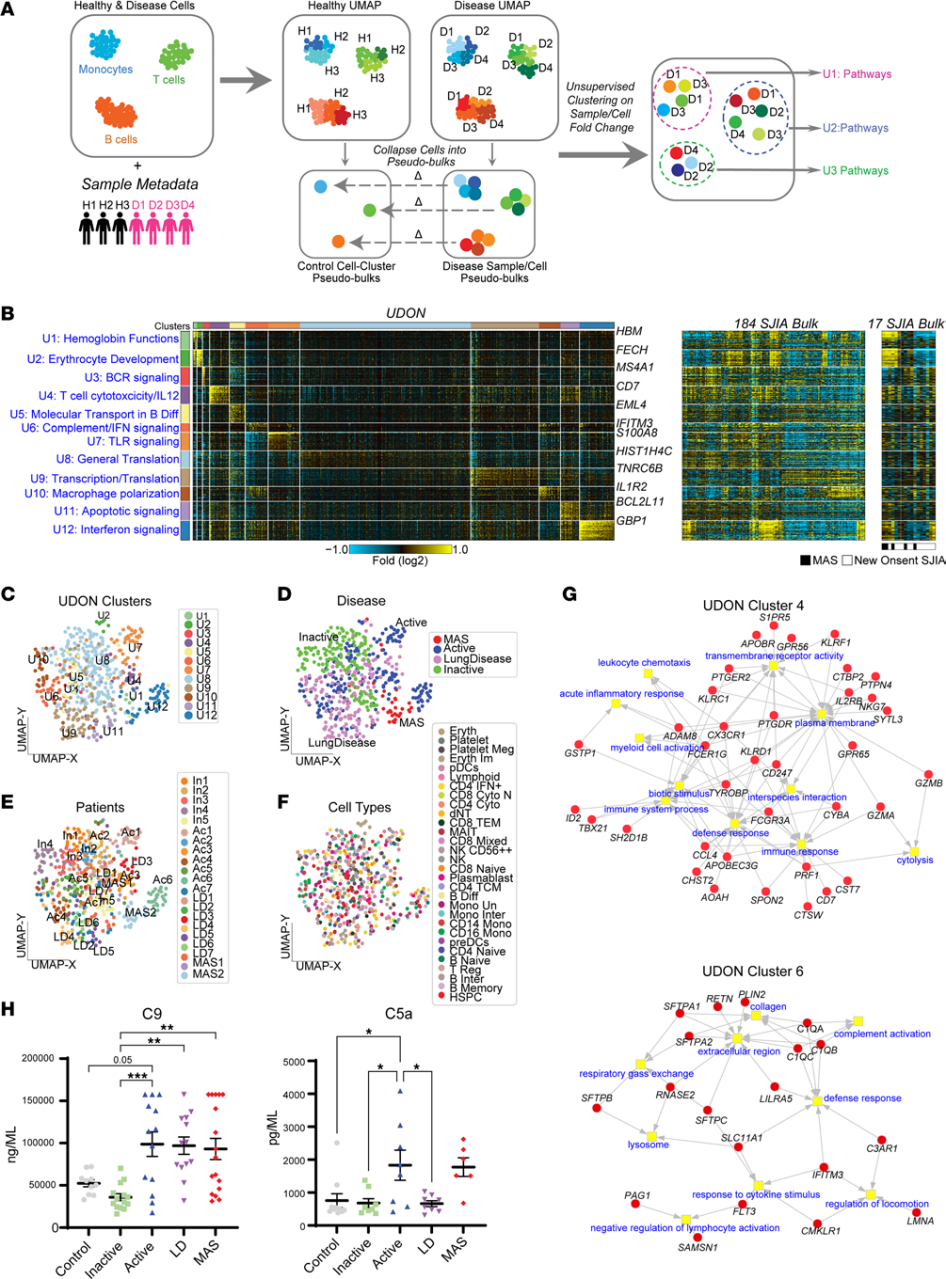
UDON analysis defines new SJIA disease subtypes including complement activation in monocytes in patients with SJIA-LD. (**A**) Overview of the UDON analysis pipeline, an unsupervised clustering method applied to control normalized patient pseudo-bulks to define disease subtypes. (**B**) SJIA UDON patient cell subtypes (UDON clusters 1–12), defined by the top cluster marker genes and top enriched pathways (PathwayCommons), are shown in the left heatmap. UDON SJIA subtypes were confirmed from 2 independent bulk SJIA transcriptomics cohorts, as shown to the right of the UDON heatmaps with matching genes, normalized to within-cohort controls. Confirmation of UDON SJIA subtypes from 2 independent large bulk SJIA PBMC transcriptomics cohorts are shown to the right of the UDON heatmap, with matching genes, normalized to within-cohort controls. (**C**–**F**) UMAP visualization of control normalized patient pseudobulks for UDON clusters (**C**), clinical subtypes (**D**), individual patients (**E**), and cell populations (**F**). (**G**) Gene-to-GO term associations (GO-Elite) for UDON cluster 4 and cluster 6. (**H**) Serum protein expression of complement component C9 and C5a of healthy controls (*n* = 10) and patients with SJIA (*n* = 57) by ELISA. Error bars indicate mean SD. Significant differences calculated by 1-way ANOVA (*P_adj_ ≤ 0.05, **P_adj_ ≤ 0.005, ***P_adj_ ≤ 0.001).

The above findings, including IFN-pathway activation, monocyte activation, and changes in the T cell compartment, provide important insights into the broad pathogenesis of SJIA. However, given the significant patient-level clinical heterogeneity in SJIA (Figure 1B), we reasoned that clinical disease groups such as active, inactive, and MAS may be largely arbitrary, with respect to the underlying biology. To address this limitation, we developed an unsupervised strategy to uncover de novo shared transcriptional programs and patient subtypes that extends to cell-type level. Rather than focus on the individual cells, our approach, called UDON, specifically considers transcriptomic differences for each patient cell-type specific pseudobulk, compared with the combination of controls for that cell type (Figure 4A). This approach collapses gene expression for all cells in a cell population into a single vector. As this vector is normalized against the average gene expression profile of all healthy matched controls for the same cell type, only patient-specific disease patterns should emerge. Unsupervised clustering of these control normalized patient pseudobulk differentials is performed in the software ICGS2 to find shared disease-specific gene expression programs that emerge from all cell populations, patients, and genes (Methods) (22, 23). To inform underlying biology, UDON reports the dominant impacted pathways for each discovered UDON gene cluster (Figure 4A). In contrast with other approaches, such as covarying neighborhood analysis (CNA), that identify gene modules that covary across samples, UDON identifies pseudobulk clusters and their most discriminate markers (4). When applied to our SJIA cohort, UDON found 12 distinct clusters, denoted U Clusters (U), which include those enriched in type I and II IFN signaling (U12), T cell cytotoxicity/IL12 (U4), Erythrocyte Development (U2), and Macrophage polarization (U10). Importantly, we were able to confirm the existence of all UDON clusters by using bulk transcriptomes of 2 independent SJIA cohorts (201 patients and 53 people in the control group (13, 24), which demonstrate these signatures in subsets of patients. (Figure 4B, Table 1, Supplemental Figure 12, and Supplemental Tables 5 and 6). While UDON clusters were derived from a small number of patients with SJIA-MAS, bulk PBMC transcriptomes from previously reported patients with MAS (*n* = 5) (13) further display the same enrichment of type I and II IFN signaling (U12). Finally, using CNA, we identified 7 out of the 12 UDON clusters, based on the comparison of top correlated genes from CNA’s reported top 10 principal components (Supplemental Figure 13, Supplemental Table 7, and Methods), further supporting the validity of UDON clusters. As UDON is a fully unsupervised approach with no prior imposed gene sets, a potential limitation is that individual UDON clusters can be composed entirely of pseudobulks derived from only one patient. Nonetheless, we observed no UDON clusters derived from a single patient or from only one disease group, and cell types did not exclusively group together in the same UDON clusters (except U5), indicating clusters are driven, rather, by gene programs expressed across cell types (Figure 4, C–F). To confirm that UDON results are stable with different target clustering resolutions or fewer samples, we tested a range of clustering resolutions and a reduced data set of patients and controls (Methods). These analyses demonstrate that UDON is highly consistent when most patients that comprise an UDON cluster are present (Supplemental Table 8 and Supplemental Figure 14).

Two UDON clusters, U4 (T cell cytotoxicity/IL-12) and U6 (complement induction), which result in distinct inflammatory pathways unique to active and SJIA-LD (Figure 4G)were particularly surprising in our analysis. Specifically, U6 was intriguing as it was consistently induced in monocytes and pre-DC from all patients with SJIA-LD, including complement genes *C1QA-C* along with *IFITM3* and *FLT3*. This cluster was further characterized by induction of surfactant genes *SFTPA1*, *SFTPA2,* and *SFTPB*, which is particularly striking given the association of U6 with SJIA-LD pseudobulks (FDR-adjusted *P* value < 0.1) and the histologic finding of dysregulated surfactant processing and PAP in such patients (7) (Table 1, Methods). Complement activation has not been well described in SJIA pathogenesis; thus, we performed an external validation for complement components in serum from patients with SJIA (Figure 4H, Supplemental Figure 15). Using ELISA, we found significantly elevated levels of C9 in patients with SJIA-LD, SJIA-MAS and active SJIA compared with people in the control group or patients with inactive SJIA. Significantly elevated levels for C5a were also observed for patients with active SJIA versus people in the control group and people with inactive SJIA, while C4 was elevated by trend in patients with SJIA-LD and SJIA-MAS patients compared with people in the control group (Figure 4H and Supplemental Figure 15), supporting that enhanced monocytic and macrophage production of complement represents a high value target for further research.

### Identification of new cellular phenotypes associated with de novo SJIA subtypes using SATAY-UDON.

**Figure 5:**
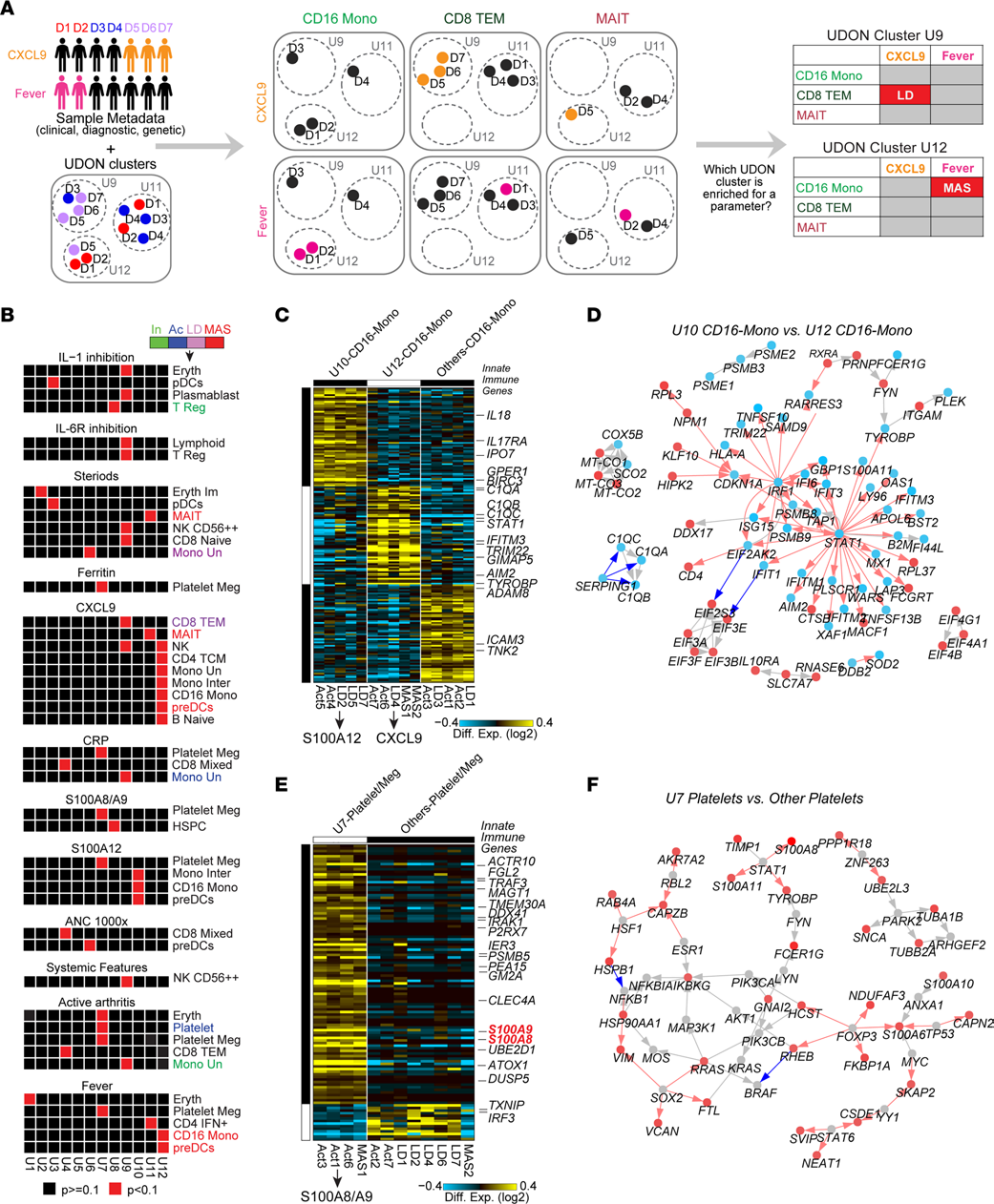
SATAY-UDON reveals novel cytokine and IFN signaling networks in SJIA-MAS nonclassical monocytes. (**A**) Overview of the SATAY-UDON analysis. Associations between UDON clusters and sample metadata (disease group, clinical parameters, or treatment) are assessed for each cell type. (**B**) SATAY-UDON results depicting associations of UDON clusters with treatments or clinical parameters and cell-types. Cell types are colored (green, inactive SJIA; blue, active SJIA; lilac, SJIA-LD; red, SJIA-MAS) if also associated to a patient group. (**C**) Revised MarkerFinder analysis and (**D**) DEG network (*P* ≤ 0.05) comparing gene programs of CD16 mono pseudobulks of U10 and U12 versus CD16 mono in other UDON clusters. (**E**) Marker genes and (**F**) impacted genes of platelet megakaryocyte pseudobulks aligning with the gene program of U7 versus platelet megakaryocytes in other UDON clusters.

While UDON clusters provide intriguing putative insights into cellular and patient heterogeneity with disease, to understand complex phenotypic associations, we require methods to link clinical and diagnostic assay metadata with these predictions. To solve this challenge, we developed an accessory approach called SATAY-UDON. SATAY-UDON considers phenotypes (e.g., disease classification and histology) and molecular correlates (e.g., metabolic readouts) together with nonredundant donor and cell-type associations in each UDON cluster using a metadata enrichment protocol (Figure 5A, Methods). Applied to our SJIA cohort, SATAY-UDON identified 40 phenotype-to-UDON cluster associations, suggesting patients represented in the UDON clusters share underlying clinical or diagnostic features (Figure 5B and Supplemental Figure 16). A subset of these SATAY-UDON associations is also identified in the top 5 expanded cell types in CNA’s phenotypic association tests (Supplemental Figure 13C, Methods). Importantly, nearly all of these observations were unique to UDON clusters as opposed to clinically defined SJIA subtypes.

SATAY-UDON finds a bias toward patients treated with steroids in UDON cluster U2, which contained genes involved in Erythrocyte differentiation (e.g., *FAM10B, FECH, BPGM,* and *AHSP*). All U2 were immature erythrocytes from 3 active SJIA, 3 SJIA-LD and 1 SJIA-MAS samples. In contrast, U1 was associated with fever, which was also comprised of erythrocytes (and platelets) and enriched in genes encoding hemoglobin subunits as opposed to differentiation (Supplemental Figure 17 and Table 1).

In UDON cluster U4, we observed a strong association with high absolute neutrophil count (ANC) and higher C-reactive protein (CRP), well accepted markers for high underlying disease activity in SJIA (Figure 5B and Supplemental Figure 17). U4 consists primarily of cytotoxic T cells and CD4 and CD8 mixed cells from 3 different active SJIA samples and 5 SJIA-LD samples. U4 marker genes are enriched in IL-12 mediated signaling and predicted cytotoxicity associated genes (e.g., *KLRF1, PRF1,* and *CCL4*) (Supplemental Figure 17 and Table 1).

Intriguingly, UDON cluster U12 showed associations of both fever (with CD16 Monocytes and pre-DCs) and CXCL9 secretion (several myeloid and lymphoid populations), comprising all patients with MAS plus one patient defined as active SJIA but noted to have subclinical MAS, further validating the clinical connection between these patients. U12 was principally associated with type I and II IFN signaling, with IFNγ as the central driver of CXCL9 secretion (16). A more focused transcriptional analysis of CD16^+^ monocyte pseudobulks found 3 different CD16^+^ monocyte transcriptional phenotypes associated with distinct inflammatory and RNA-binding pathways, which subdivided patients with active SJIA, MAS, and SJIA-LD into novel subsets. We identified an expanded U12 cluster of patients with active SJIA, MAS, and SJIA-LD, as well as a U10 cluster of CD16^+^ monocyte pseudobulks from patients with active SJIA, MAS, and SJIA-LD, both of which were distinct from other CD16^+^ monocyte pseudobulks. The U10 cluster, defined by marker genes *IL1R2* and *CD163*, was associated with elevated S100A12 serum levels. The U12 cluster was associated with elevated CXCL9 serum levels and upstream of both type I and II IFN pathway genes, we observed upregulation of a network of key inflammatory transcription factors previously implicated in IFN regulation (*STAT1* and *IRF1*) (Figure 5, C and D). This further supports a central role of CD16 monocytes in driving the IFN response in SJIA-MAS.

Finally, UDON cluster U7, enriched in TLR signaling among platelets and megakaryocytes was found to be associated with elevated serum levels of S100A8/A9 and S100A12. Notably, *S100A8* and *S100A9* mRNAs were the principal markers of this population. Unsupervised analysis of these platelet pseudobulks found an expanded U7 cluster comprised of 3 patients with active SJIA and 1 patient with MAS, enriched in genes involved in the induction of apoptosis (e.g., *NOTCH2* and *TNFSF10*) and NF-κB signaling (e.g., *IRAK1* and *TRAF3*) (Figure 5, E and F). Given emerging data that S100 proteins signal through TLR (25), these findings could indicate a novel mechanism where platelet-mediated activation via S100 proteins drives inflammation in SJIA. Hence, these findings support the notion that heterogenous immunological diseases with variable clinical features and biomarkers may stem from identifiable cell-type–specific disease subtypes.

### UDON clusters represent broadly conserved transcriptional programs across a pan-immune landscape.

**Figure 6:**
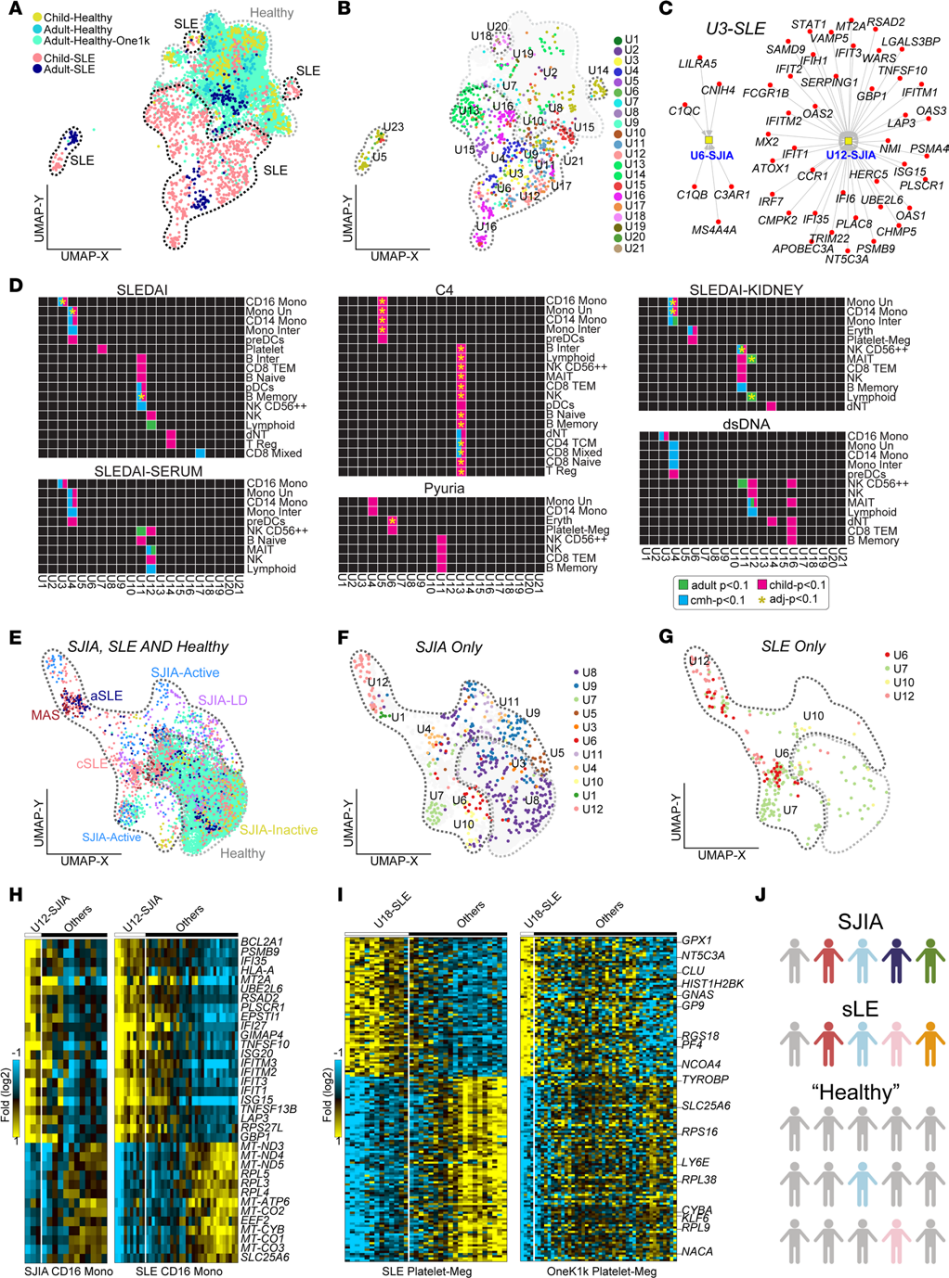
UDON analysis identifies broad subtypes and inflammatory programs in a pan-immune atlas. (**A**) Joint UMAP of SLE cohort (adult, child, controls) with healthy PBMC samples in the OneK1K cohort (pseudobulk folds). The drawn boundaries in the UMAP designate control enriched populations (grey) versus SLE (black). (**B**) Visualization of only SLE samples from panel **A**, colored according to the UDON cluster number. (**C**) Marker genes in SLE-UDON cluster 3 (*n* = 60) that overlap with enriched SJIA-UDON clusters 6 (complement activation) and 12 (interferon signaling). (**D**) Age-independent and age-specific SATAY-UDON results for selected SLE clinical parameters (blue, *P* < 0.10 CMH test; green, *P* < 0.10 adult-specific; pink, *P* < 0.10 child-specific 1-sided Fisher’s Exact Test; yellow star, FDR-adjusted *P* < 0.10)). (**E**) Joint UMAP of SJIA, OneK1K and SLE samples (each dot is a normalized pseudobulk) for SJIA UDON marker genes, colored by the known clinical subtype of each sample. The drawn boundaries in the UMAP designate control-enriched populations (grey) versus systemic inflammatory disease (black). (**F**) Visualization of only SJIA samples on the same UMAP as panel **E**, colored according to UDON cluster number from Fig. 4C. (**G**) Same UMAP as panel **E** but highlighting the SLE samples, colored by projected SJIA-UDON cluster labels from panel **F** onto SLE samples. (**H**) Heatmap of common DEGs (fold > 1.2 and empirical Bayes *t* test *P* < 0.05, 2 sided), for SJIA UDON cluster 12 CD16 monocyte pseudobulk folds versus other CD16 monocytes in the SJIA cohort and SLE cohort. (**I**) Heatmap of common DEGs (fold > 1.2 and empirical Bayes *t* test *P* < 0.05, 2 sided), for SLE UDON cluster 18 platelet pseudobulk folds versus other platelets in the SLE and OneK1K cohort. (**J**) Proposed model for disease heterogeneity among systemic inflammatory disease patients and presumably healthy donors in the populations.

To determine if these disrupted signaling networks are unique to SJIA or shared across other systemic inflammatory disorders, we performed a comprehensive pan-immune survey of de novo subtypes, leveraging existing single-cell profiles from 41 patients with SLE and 982 healthy donors (OneK1K cohort) (2, 3). We first performed UDON on a previously reported cohort of 33 patients with childhood-onset SLE (cSLE), as well as 8 patients with adult-onset SLE (aSLE) with matched controls (2). Using our 30 PBMC cell populations as a common reference, we produced pseudobulk folds for each child and adult relative to their matched controls. To assess the disease significance, we also produced pseudobulk folds for all healthy controls, relative to their collective average (Figure 6A, Methods). When applied to these SLE pseudobulk differentials, UDON found 21 stable clusters, after considering a range of possible resolutions (Figure 6B, Methods). While most of the UDON clusters were primarily composed of pediatric cases, only 3 (U5, U16, and U19) were unique to cSLE, and no clusters were unique to a single patient (Supplemental Figure 18). Notably, 8 of the SLE-UDON clusters mapped to at least 1 SJIA-UDON cluster, with most of the remaining mapping to SJIA cell and/or subtype signature (e.g., CD16 monocytes in SJIA-LD) (Supplemental Figure 18 and Table 2). Further analysis of SLE UDON cluster U3 showed that this CD14/CD16 monocyte–dominated cluster corresponded to our complement-associated SJIA-LD–enriched cluster U6 and type I and II IFN-associated SJIA-MAS–enriched U12, based on gene set enrichment (Figure 6C and Supplemental Figure 18).

To examine the associations of UDON clusters with markers of SLE disease activity, we used SATAY-UDON with the associated SLE clinical metadata (2), which replicated earlier findings as well as proposed new disease associations (Figure 6D, Supplemental Figure 19, and Supplemental Table 9). For these analyses, we employed both the CMH procedure to account for covariate association differences among adult and pediatric patients in addition to the standard Fisher Exact test, applied separately to each age group (Methods). Prior work with this cohort demonstrated a strong IFN signature across several cell types and associated abundance of those clusters with higher cSLE disease activity (2, 19). In support of this, we found that SLE UDON clusters that map to IFN signaling (SLE-U3 and SLE-U11) (Supplemental Figure 18) in CD16 Monocytes and B Memory cells were associated with high SLE disease activity index (SLEDAI) scores, as well as in NK cells associated with kidney involvement (adjusted *P* < 0.1, CMH). More age-specific associations of IFN signaling UDON clusters, U3 and U11, were observed with dsDNA levels, serum subscore of the SLEDAI, and pyuria (Figure 6D).

Strikingly, we also found a previously unrecognized association of higher complement component C4 levels in children with SLE-U13, which maps to IL-6 mediated signaling events, and this association was driven by a subset of lymphoid cell types, including NK, CD8 mixed, and regulatory T cells. C4 was similarly associated with IL-5 mediated signaling SLE-U5 cluster, driven by monocytic cell-types (adjusted *P* < 0.1 for CMH test) (Figure 6D). These findings are notable as they nominate gene pathways that may be relevant in patients with lupus without hypocomplementemia. We further identified associations of specific treatments such as hydroxychloroquine with SLE-U18 (mapping to the clotting cascade) and other age-group–specific associations of SLE-U3 and SLE-U11 with rash, erythrocyte sedimentation rate (ESR) and arthritis in children (Supplemental Figure 19 and Supplemental Table 9).

Finally, to determine the overall pan-immune landscape of these inflammatory diseases we joint embedded SLE pseudobulk differentials and those derived from 982 healthy donors from the OneK1K cohort into a SJIA-centric UMAP via UMAP projection. Considering all 30 cell types, we show broad alignment of controls from all 3 cohorts with inactive SJIA as well as alignment of SJIA-MAS with aSLE (Figure 6, E and F, Methods). Projecting labels from SLE to SJIA pseudobulk differentials, we found that the monocytes of a subset of cSLE patients phenocopy the SJIA macrophage activation (SJIA-U12) (Figure 6, G and H). While the OneK1K cohort presumably comprises only healthy controls, it was previously discovered that known and novel autoimmunity-quantitative trait loci associated with distinct autoimmunity implicated regulators in specific cell types (3). Given these results, we projected all healthy control pseudobulk differentials into both SJIA and SLE UDON clusters. Inspection of these results found that multiple SLE UDON subtypes were assigned to a small number of people in the healthy control group (Supplemental Figure 18C), including intermediate-monocytes of complement/interferon-associated SLE-U3 and fibrin clotting-associated platelets from SLE-U18 (Figure 6I and Supplemental Figure 19C). These findings support the hypothesis that single-cell genomics can identify emerging conserved autoimmune programs in health and disease as well dominate novel diagnostic gene-regulatory programs (Figure 6J).

## Discussion

In this study, we discover subtypes of systemic inflammatory disease that selectively associate with clinical features and biomarkers. To overcome existing analytical limitations, we defined a computational approach to define subtypes that redraw established clinical classifications through transcriptomics at the level of individual cell types. These data suggest the existence of conserved, prespecified gene programs within the same or similar cell types in distinct systemic inflammatory disorders. Our focused analysis of SJIA revealed distinct impacted inflammatory pathways that resolve different stages of disease, including active disease, inactive disease, and MAS. Although prior work has identified potential pathogenic programs in SJIA including monocyte, neutrophil, and T and B cell activation (12, 20, 26–29), the underlying causes and contributors to this heterogeneity remain unknown. Here, our initial analysis revealed activation of IFN-related genes across multiple cell types, particularly in SJIA-MAS, distinct transcriptional changes in monocyte populations, and differences in the T cell compartment. However, given the marked patient-level heterogeneity, we developed methods called UDON and SATAY-UDON to identify and describe novel disease-associated transcriptional programs implicating what we believe to be new potential drivers of SJIA pathogenesis, including IFN activation, multiple distinct monocyte phenotypes, and platelet activation. Critically, we found that many of these transcriptional programs are broadly conserved across the immune landscape and represent previously hidden drivers of inflammatory diseases.

By identifying patient subclusters at the individual cell-population level, UDON can overcome an inherent limitation of existing supervised comparison methods. A principal aim of single-cell genomics analysis is the ability to not only resolve cell populations, but also complex underlying disease programs. UDON exploits well-established integration and single-cell clustering approaches to define patient-specific differences that underlie hidden disease programs. While the number of donors may be limited within this cohort, we orthogonally confirmed the presence of UDON signatures using independent bulk transcriptomic cohorts of SJIA, CNA, and experimental validations. SATAY-UDON then further illustrates how these previously hidden programs are associated with clinical measures. Together, UDON and SATAY-UDON predictions provide new insights, such as the predominance of IFN-driven activation in SJIA-MAS and a monocytic-driven complement and interferon phenotype in SJIA-LD.

Critically, we found that UDON clusters represent not just features of SJIA pathogenesis, but broadly conserved transcriptional programs present across inflammatory disease states. We identified homologous UDON clusters present in both adult and childhood-onset SLE, with UDON clusters associated with distinct clinical and serological markers of lupus disease activity. Projecting transcriptomic data from over 1,000 healthy donors and patients with SLE across UDON clusters, we demonstrated that healthy individuals largely diverge from those with inflammatory disorders. However, a minority of healthy individuals have UDON clusters that cosegregate with those from patients with SJIA and SLE. It is tempting to speculate that such individuals have subclinical disease or underlying genetic predisposition to autoimmunity.

An important example is a new UDON-SJIA cluster dominated by IFN signaling (U12) present in patients with clinical evidence of overt and subclinical MAS, expressed by CD16^+^ and intermediate monocytes, pre-DCs, and other lymphocytic cell types. SATAY-UDON analysis associated this UDON cluster with elevated levels of CXCL9, an IFNγ-induced chemokine and specific MAS biomarker (16). These findings align with our demonstration that the most distinct IFN signature was expressed by monocytic and CD4 IFN^+^ cells from patients with SJIA-MAS, with a lesser degree of elevated expression of these genes for monocytes from active SJIA and SJIA-LD. Prior work showing that monocytes from patients with SJIA and patients with untreated MAS or with secondary HLH (sHLH) are hyperresponsive to IFNγ further supports the hypothesis that this IFN signature driving MAS is derived from monocytes (20, 30, 31).

Our analysis also revealed that distinct monocyte transcriptional programs exist across the SJIA disease spectrum, including a myeloid polarization cluster (U10) expressed by patients with active SJIA and SJIA-LD and associated with elevated S100A12 levels. The transcriptional profiles of CD16^+^ monocytes in this UDON cluster showed marked differences from those of other patients, including high *CD163* expression, a myeloid differentiation marker that is responsible for binding and engulfing hemoglobin-haptoglobin complexes. Intriguingly, elevated *CD163* expression has also previously been identified as a marker for patients who fail anti–IL-1 therapy, further highlighting how different monocyte phenotypes link to disease biology (14).

UDON discovered a distinct, monocytic driven complement and interferon program (U6), which was present in patients with SJIA-LD and several patients with active SJIA. This monocyte program is particularly notable as it also shows high levels of surfactant protein expression; dysregulated surfactant metabolism in the lungs is a key histologic feature of SJIA-LD (7). SJIA-LD shares characteristics with PAP, which is defined by intraalveolar accumulation of surfactant proteins due to impaired clearance by alveolar macrophages (32). Surfactant expression in circulating monocytes, as seen here, may reflect a specific subset of blood monocytes primed to migrate into lung tissue, where they subsequently differentiate into alveolar macrophages (33). This transcriptional program was also found in monocytes of patients with cSLE and associated with markers of severe disease activity.

While prior work has found increased expression of some complement pathway genes or proteins in SJIA (34–39), to our knowledge, no study has investigated the involvement of complement in SJIA-LD. The detected increase of several serum complement proteins in patients with SJIA observed here may partially derive from hepatocytes in the liver, which are considered the predominant source of complement components (40). However, recent scRNA-Seq studies have highlighted a distinct monocyte/macrophage subset expressing *C1QA-C* in patients with pediatric SLE, Behḉet’s and Kawasaki diseases, adult Rheumatoid Arthritis, and bacterial infections (2, 41–44). In addition, a human cross-tissue scRNA-Seq study characterized a specialized lung alveolar macrophage subset strongly expressing *C1QA-C* (45), and our recent work examining lung tissue in a mouse model of MAS found a similar MAS-specific macrophage cell cluster enriched for complement activation (46). Clinically, low C3/C4, possibly reflecting complement consumption, has been described in 2 patients with SJIA-MAS and 1 patients with adult-onset Still’s disease MAS (47). Furthermore, a recent study of 23 patients with refractory HLH found 70% simultaneously present with complement-mediated thrombotic microangiopathy (TMA). These authors hypothesized that the high levels of IFNγ in HLH activates complement, which then causes endothelial injury and damage in TMA (48). Together with the findings of Zheng and colleagues, who demonstrate that the C1Q-high inflammatory monocyte phenotype present in patients with Behḉet’s disease is in vitro inducible with IFNγ (44), this highlights the potential interactions of complement activation and IFN signaling in driving pathogenesis in the SJIA clinical spectrum, and the conserved role of this monocytic program across the pan-immune landscape.

SATAY-UDON revealed an unexpected association of increased serum levels of the alarmin proteins S100A8/A9 and S100A12 with platelet megakaryocytes in UDON cluster U7, which was enriched for genes involved in TLR signaling. S100A8/A9 has been previously shown to signal through both RAGE and TLR4 to amplify inflammatory responses and is likely the key driver of the TLR signaling pathways detected in U7 (25). Platelet S100A8/A9 levels are also increased in patients with SLE and peripheral artery disease and are thought to promote thrombosis and cardiovascular disease (25, 49, 50). Such elevated platelet and platelet megakaryocyte frequencies are detected in active SJIA and MAS (51), which supports the hypothesis that, in SJIA, activated platelets may contribute to inflammation by releasing S100A8/A9 in the microenvironment to drive inflammation and thrombosis (50).

We also find a strong association with well-described clinical markers of high disease activity in SJIA — both laboratory parameters, such as CRP and ANC, and active arthritis — and cytolytic T cell populations in the IL-12 signaling/cytotoxicity cluster U4. Indeed, most cytotoxic T cell pseudobulks were associated with U4, except for those from the patients with SJIA-MAS who, rather, clustered with the IFN-driven U12. More broadly, our analysis of the T cell landscape supports a shift from more naive to more active populations in SJIA. Recent work identifying a common HLA-DRB1*15 haplotype in many patients with SJIA-LD (52) has suggested that this clinical subtype could have distinct patterns of T cell activation (21). Indeed, our analyses found that lymphocyte population in SJIA-LD have a striking transcriptional signature that has some similarity but is distinct from that seen in SJIA-MAS. However, we saw similar patterns of Th1/Th2 markers across the active disease populations. We also observed no changes in Th17 polarization, as has been previously reported (26). Intriguingly while the MAIT cells were significantly lesser in the disease group, these cells presented a strongly dysregulated gene expression profile particularly in patients with SJIA-MAS, but also in patients with active SJIA and SJIA-LD. Finally, patients with SJIA-MAS had significantly more dNTs, a cell type that was also expanded in pediatric lupus and is proposed to represent an end-stage T cell subset particularly efficient in cytokine secretion and cytotoxicity (53). While these clinical associations suggest hypotheses underlying the transcriptional programs, precise validation approaches are required in specific cell populations in patients.

In conclusion, UDON and SATAY-UDON offer a exploratory computational strategy to discover distinct transcriptional programs in large clinically heterogeneous patient cohorts. Through this approach, we find what we believe to be a previously unexplored, shared monocytic complement and IFN gene program in SJIA-LD that is also present in lupus and associated with markers of high disease activity. We also discovered a role for platelets in driving SJIA inflammation and evidence for distinct monocyte transcriptional phenotypes present across inflammatory disorders including lupus, SJIA, and MAS. Importantly, this method highlights heterogenous clinical phenotypes and serum measurements that underlie these subtypes, suggesting that transcriptional programs result in separable and durable disease responses. Together, this approach can identify diverse transcriptional programs found across cell types in severe systemic autoimmune and autoinflammatory disorders. We anticipate new opportunities to improve and expand these computational approaches in the future, including improved means to distinguish cell-type specific heterogeneous disease impacts and automatically accounting for potential batch effects in various stages of the analyses.

## Methods

### Experimental design

This was a retrospective cohort study of children with SJIA, SJIA-MAS, and SJIA-LD. Clinical and laboratory features were obtained from the electronic medical record using a standardized case report form. All patients were diagnosed with SJIA based on the International League of Associations for Rheumatology (ILAR) criteria (54); however, for newly diagnosed patients, samples were obtained and treatment initiated with a disease duration of under 6 months, using the operational definition of SJIA (55). Inactive disease was defined based on the Wallace criteria (56); conversely, patients were considered to have active SJIA if they had any active clinical features including arthritis, rash, fever, adenopathy, hepatosplenomegaly, or elevated CRP or erythrocyte sedimentation rate. Patients were diagnosed with MAS based on diagnosis of the treating physician. Patients were considered to have SJIA-LD if they had both clinical and radiographic features of LD based on definition of probable or definite SJIA-LD (1).

### Sample collection

Fresh whole blood was collected in CPT tubes by venipuncture, mixed by inverting the tube 8–10 times and then centrifuged at 1600*g* for 20 minutes at room temperature. The cell pellet containing isolated PBMCs was washed twice with PBS, resuspended at approximately 2-to-4 × 10^6^ PBMCs/mL in freezing media (90% FCS/10% DMSO) and stored in liquid nitrogen until further processing.

### Complement protein analysis

Serum levels of complement proteins were analyzed either by Luminex (R&D Systems) for C5a, C1q, C4 and MBL, by C9 ELISA (Abcam) or TCC ELISA (Mybiosource). Statistical analysis was performed with GraphPad Prism 9.3.1. Except where noted, results of statistical tests were significant when *P* < 0.05.

### PBMC isolation and single-cell RNA-Seq

Frozen PBMCs were thawed and washed twice with PBS. Dead cells were removed with a dead cell removal kit following the manufacturer’s instructions (Miltenyi Biotec), and then resuspended at 700–1,200 cells/μL in PBS (total count 12,800 cells). Samples were then sequenced with 10× Genomics (Pleasanton), version Chromium NextGEM (Chemistry: 3′v3 Assay)

### scRNA-Seq analysis

Details on read alignment, cell clustering, differential gene expression analyses, and supervised clustering are provided in the Supplemental methods section.

### UDON

#### Intuition.

UDON is an unsupervised approach to detect novel gene regulatory programs that are common or different among heterogeneous disease samples based on scRNA-Seq. We assume that prior defined clinically annotated disease subtypes are imprecise, with the goal of redefining them at a pseudobulk fold level. UDON leverages the common concept of a pseudobulk profile, which is the average gene expression of a set of cells (usually from a cell type or a cluster) from a donor, patient, or sample. For larger single-cell disease cohorts, individual patients can have varying cell-type frequencies. Combining all cells together, without explicitly considering which cells are associated with which patients, can lead to disease observations driven by a single patient; however, pseudobulks provide an effective way around this. The central premise of this approach is that, once cell types are defined from a cohort (outside of this program), case and control cohort designs can be exploited to identify common gene expression responses between sample-level gene expression changes in individual cell populations, as opposed to single cells. Here, populations-level effects within a sample are defined by first calculating pseudobulks. Specifically, for the controls within a cohort, an aggregate pseudobulk for a cell population is calculated across all controls. If different controls exist for different patients, based on age, batch, or other factors (referred to here as batch), batch-specific pseudobulks are computed. UDON computes fold change between patient-specific pseudobulks compared with the aggregate controls for each cell type. In special scenarios, such as the analysis of the OneK1K cohort, pseudobulk fold changes are also computed for each control donor and cell type. The fold change is calculated between the sample-specific pseudobulks against the aggregate control pseudobulks. We cluster pseudobulk fold change instead of pseudobulks, as the latter alone would only identify distinct cell-type clusters, as opposed to common disease-specific transcriptional differences. Using the pseudobulk fold changes from samples with disease, the software finds clusters of pseudobulks with common gene expression changes by applying sparse nonnegative matrix factorization–based (NMF-based) clustering called ICGS2. ICGS2 is an unsupervised method to define populations from bulk or scRNA-Seq in AltAnalyze and defines clusters using iterative guidegene focused sample/cell clustering to find coherent correlated gene modules. UDON is thus fully unsupervised and independent of prior gene sets and patient subtypes. An UDON cluster can be composed entirely of normalized pseudobulks from one cell type or many. It is a naive subtype identification approach that is only dependent on the initial cell-type definitions and the selection of explicit control samples. This program can identify patient populations with or without a preimposed k-parameter (desired number of clusters), but only reports NMF-defined clusters with unique marker gene expression. For ICGS2, we used the default program options with the additional parameters ρ set to 0.3, markerPearsonCutoff set to 0.2, *k* set to a range of tested values (10–30), and FoldDiff set to 2. A final k resolution of 15 was selected in SJIA and 25 for SLE, as the number of predicted clusters beyond these numbers failed to substantially increase the number of stable clusters. For each cluster, unique marker genes were determined using the MarkerFinder module of AltAnalyze. Table 1 displays the top 10 unique ranked marker genes using this MarkerFinder statistic.

#### Assumptions.

We make the following assumptions before applying this algorithm: (a) A sufficiently sized cohort of patients is required to identify shared gene expression programs among 2 or more patients. (b) A relatively homogenous set of controls is needed. (c) Gene expression data sets have undergone quality control (including batch effect removal) and have been log-scaled. (d) Cells have a cell-type annotation assigned manually or from an external program. (e) Phenotypically distinct cells comprise a reasonable subset of those in at least one cell type from a patient. (f) (If applying SATAY-UDON) There is at least 1 quantifiable clinical measurement for at least 2 disease samples included in the UDON analyses and the clinical measurement is transformed into a binary variable.

#### Defining sample-level gene expression change between diseased and control samples.

Consider a cohort of *d* number of disease samples and *h* number of healthy samples. Let *D* and *H* be the gene expression matrices for disease and healthy samples respectively such that *D_i_* is a *m x n_i_* matrix of gene expression, where *m* is the number of genes and *n_i_* is the number of cells for disease sample *i* ∈ [1, *d*] and *H_i_* is a *m x n_i_* matrix of gene expression, *i* ∈ [1,*h*] 
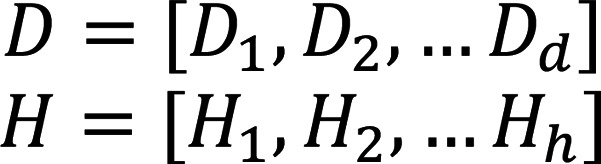


Let *j* ∈ [1, *c*] where *j* is a cell type belonging to *c* cell types assigned to the cells of the samples. Then, for a disease sample *i* ∈ [1, *d*]
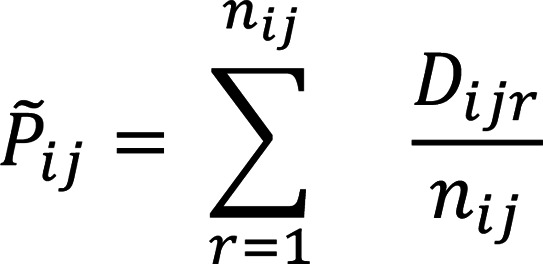


Where 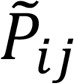 is a *m* dimensional vector representing the sample-level disease cell-type pseudobulk from *n_ij_* cells to capture sample-specific gene expression programs.

The control samples, however, are assumed to be homogenous in UDON, and, therefore, the cell-type pseudobulk *P_j_* (a *m* dimensional vector) is computed as an aggregate value across all controls. It is defined as:
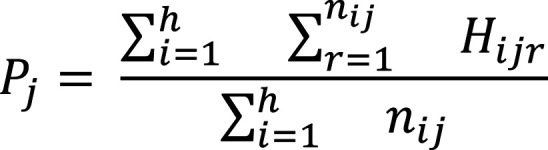


The fold change between the disease sample and the controls, for all *j* ∈ [1, *c*], is simply defined as:
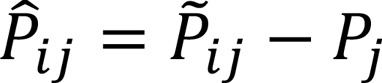


Where 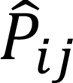 is a *m* × *c* matrix.

Lastly, prior to applying the sparse NMF clustering algorithm to the pseudobulk profiles, we ensure that 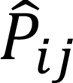 has nonnegative entries, a required condition for the algorithm. Now,
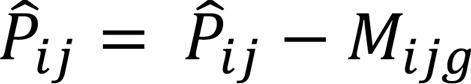


where *M_ijg_* is the minimum value of 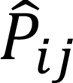 for gene *g* ∈ [1, *m*].

#### Applying sparse NMF for clustering pseudobulk profiles.

Let [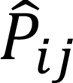] be the matrix be the collection of 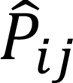 vectors for all *i* ∈ [1, *d*] and *j* ∈ [1, *c*]. We provide [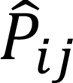] as the input for ICGS2, which applies the sparse NMF algorithm to cluster the pseudobulk profiles (the columns of [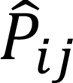]). UDON provides an optional cluster resolution parameter *k* for NMF analysis that allows users to explore broader or more granular clusters.

For the downstream analyses presented in the paper, we have considered UDON clusters derived with *k* set to 15. The resolution of 15 was selected as stable (consistent with higher resolution results) after considering k = 10, 15, 20, or 30, which reported 10, 12, 14, and 17 final UDON clusters, respectively. Dominant impacted pathways (pathway commons by default) are reported for each UDON cluster, which represent common or heterogenous groups of patients and cell types.

Statistics

To discover underlying clinical or phenotypic associations from UDON clusters, SATAY-UDON provides a statistical phenotype enrichment protocol that considers nonredundant donor and cell-type associations per UDON cluster. Our null hypothesis is that there is no association between a sample’s clinical condition and the sample’s pseudobulk profile in a gene response program (as indicated by an UDON cluster). We test this hypothesis for positive enrichment by performing a 1-sided Fisher’s Exact test on all pseudobulk profiles for a given cell-type in an UDON cluster. Fisher’s exact test requires categorical data, and thus, the continuous clinical variables are transformed into a binary variable based on clinical expert-set thresholds. This option is not provided in SATAY-UDON and must be determined by the user outside of the program. For a given cell type and clinical measure, in a 2-by-2 contingency table, let the number of pseudobulks associated with samples with the clinical condition in and not in UDON cluster U be *Q* and *r,* respectively. Similarly, let the number of pseudobulks associated with samples that do not have the clinical condition in and not in UDON cluster U be and respectively. Then, SATAY-UDON applies Fisher’s exact test on the described contingency table.

By default, 4 or more samples are required for an association to take place between an UDON cluster and clinical covariate, indicated by *Q* value in the contingency table. For a cell type, an association between a UDON cluster and a clinical variable is considered positive and visualized if the 1-sided *P* value < 0.1 for the above-mentioned Fisher’s exact test; however, SATAY-UDON provides the confidence level (*P* value) as a user provided parameters for users. We report the FDR adjusted *P* value by applying the Benjamini-Hochberg adjustment to the *P* values from each clinical variable and visualizing the associations with an FDR adjusted *P* < 0.1.

#### CMH test for confounding variables.

To protect SATAY-UDON associations against confounding variables (such as age, sex, etc.), we employ the CMH procedure to produce stratified estimates of association between UDON clusters and clinical covariates. The CMH test stratifies the samples by the categories in the confounding variable (for example, male and female if sex is a confounding variable) and considers a series of 2-by-2 contingency tables of the binary predictors for each stratum. In SATAY-UDON, for each stratum, like the Fisher’s Exact Test, the *i*th 2-by-2 contingency table, where *i* ∈ [1,c] and is the stratum in a list of categories of a confounding variable, *c* categories has the following values defined in same way as mentioned above: *Q_i_ r_i_* indicate the number of pseudobulks associated with samples with the clinical condition of interest in and not in UDON cluster, respectively, and *S_i_ t_i_* indicate the number of pseudobulks associated with samples without the clinical condition of interest in and not in UDON cluster, respectively.

By default, 2 or more samples are required in each stratum for an association to take place between an UDON cluster and clinical covariate, indicated by value in the contingency table. CMH calculates a *P* value and an odd ratio that represents a weighted association between a UDON cluster and a covariate across the strata. We report the FDR adjusted *P* value by applying the Benjamini-Hochberg adjustment to the *P* values from each clinical variable and visualizing the associations with an adjusted *P* < 0.1. For the SLE data set, we applied the CMH test, considering age as the confounding variable, to identify the associations between a UDON cluster and clinical covariate that are protected against adult and pediatric data imbalances. At least 2 samples are required in each age group of samples (also referred as stratum) for the binary predictors, UDON cluster and clinical covariate, to be considered for the CMH test. For determining age group–specific associations, we performed SATAY-UDON using the Fisher’s Exact Test on only age group–specific samples and reported the raw and FDR-adjusted *P* values. We note that other study designs may necessitate distinct testing procedures and batch effects correction beyond the parameters described here.

### CNA

Details on CNA are provided in the Supplemental methods.

### External bulk and scRNA-Seq data set analyses

Details on external SJIA and SJIA-MAS data set analyses are provided in the Supplemental methods.

### Study approval

This study was approved by the CCHMC IRB (IRB no. 2018-2408) and written informed consent was obtained from each parent or guardian. Child assent was obtained where appropriate.

### Data availability

The UDON workflow is composed of independent python and R modules. Scripts for preprocessing, data normalization, and clinical covariate association analyses (SATAY-UDON) are available on Github (https://github.com/kairaveet/udon-sjia-sle; branch name: main; commit ID: 6eeb5c35cb5ae7893ea9b33e0dae7008f05ee0c0). Unsupervised iterative clustering via guidegene selection are accomplished through the existing python2 module ICGS2 in AltAnalyze (http://www.altanalyze.org and https://github.com/nsalomonis/altanalyze; branch name: master; commit ID: 64f77a77fb3c947bd5eaaaf759bfac2ab0a1b498). An excel file indicating supporting data values for figures is provided in addition to the Supplemental tables. The processed PBMC SJIA scRNA-Seq and associated metadata have been deposited in the Gene Expression Omnibus (GEO) database (GSE207633). Raw sequencing data is available in dbGAP, associated with this GEO study.

## Supplementary Material

Supplemental data

Supplemental tables 1-9

Supporting data values

## Figures and Tables

**Table 1:**
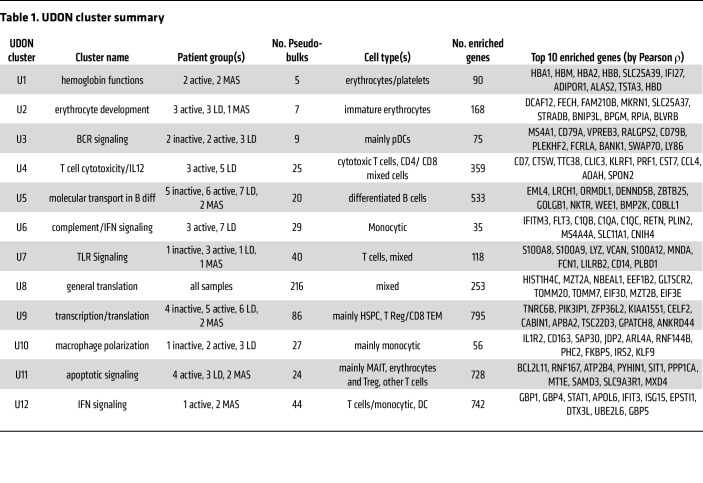
UDON cluster summary

**Table 2:**
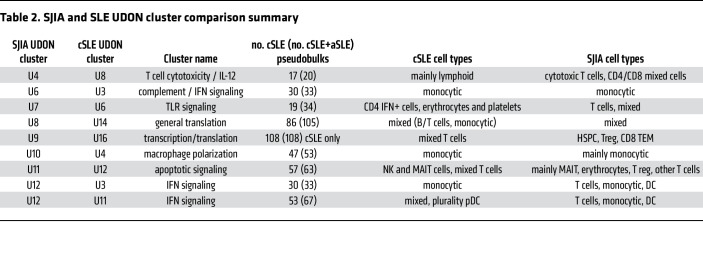
SJIA and SLE UDON cluster comparison summary

## Version 1 09/21/2023

In-Press Preview

## Version 2 11/15/2023

Electronic publication
