# Supplementary material for: Population-level single-cell genomics reveals conserved gene programs in systemic juvenile idiopathic arthritis
Source: J Clin Invest. 2023 Nov 15;133(22):e166741. doi: 10.1172/JCI166741 (PMC10645394; doi:10.1172/JCI166741)
Supplement: Supplemental data [file jci-133-166741-s076.pdf]

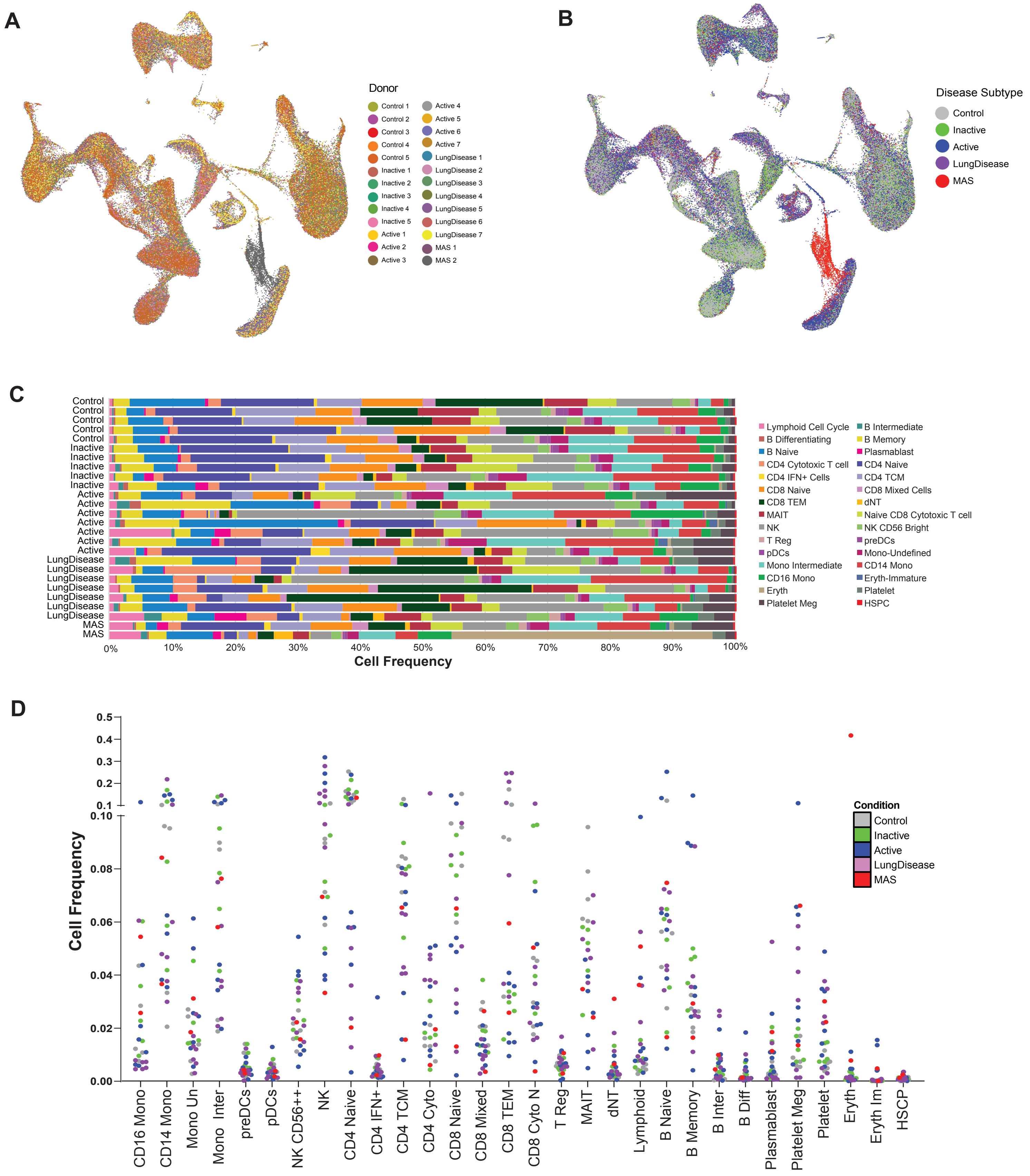

Supplementary Figure 1. Cell type distribution across donors and disease subtypes. UMAP of all cells showing distribution of A) donors and their B) disease subtypes. C) Cell frequencies per patient and per cell type including Erythrocytes. D) Cell frequency of individual patient or control sorted by cell type. Colors indicate patient subtype or control.

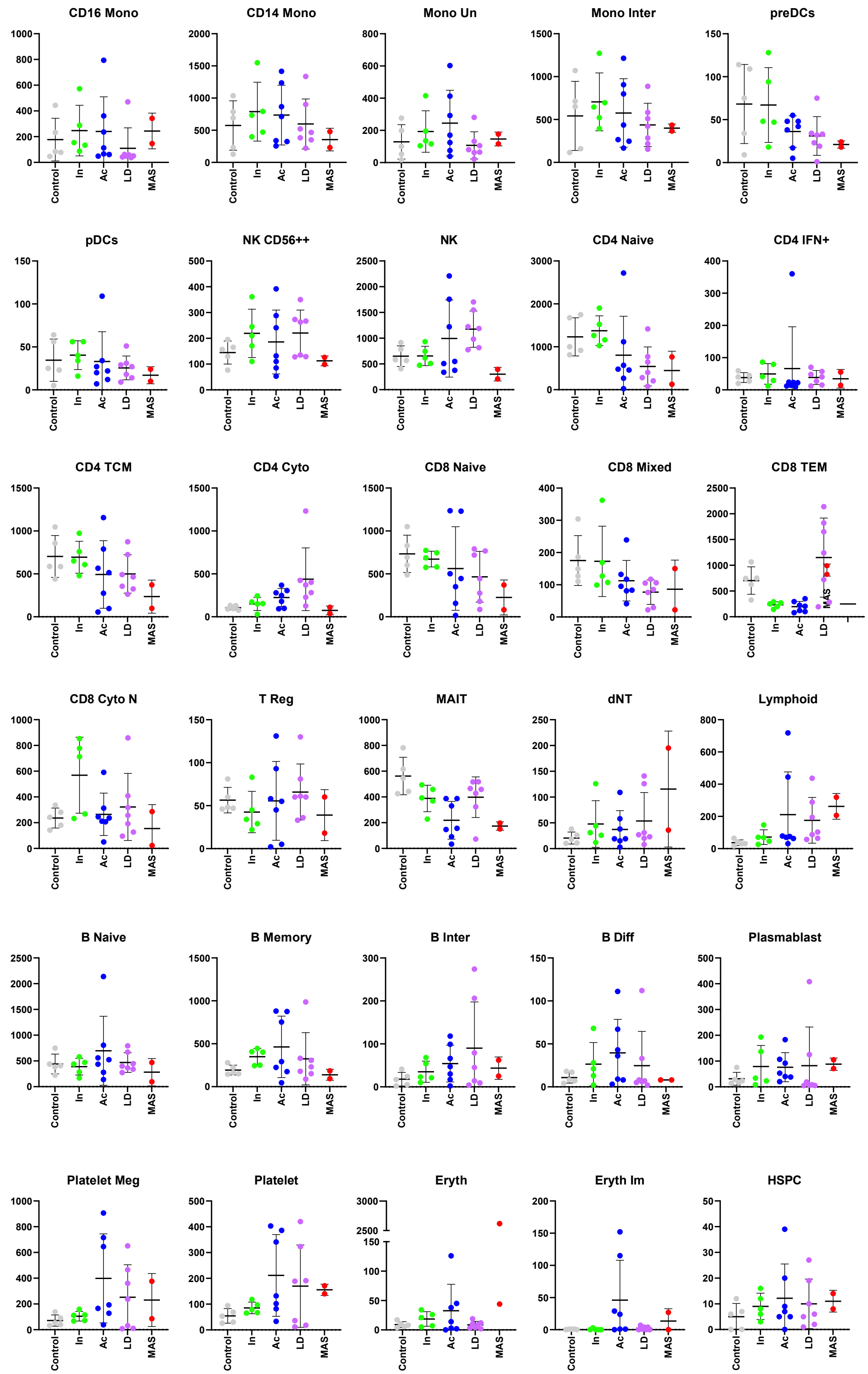

Supplementary Figure 2. Cell counts. Individual cell counts for each patient in each disease group or healthy individual in the control group, depicted separately for each cell type.

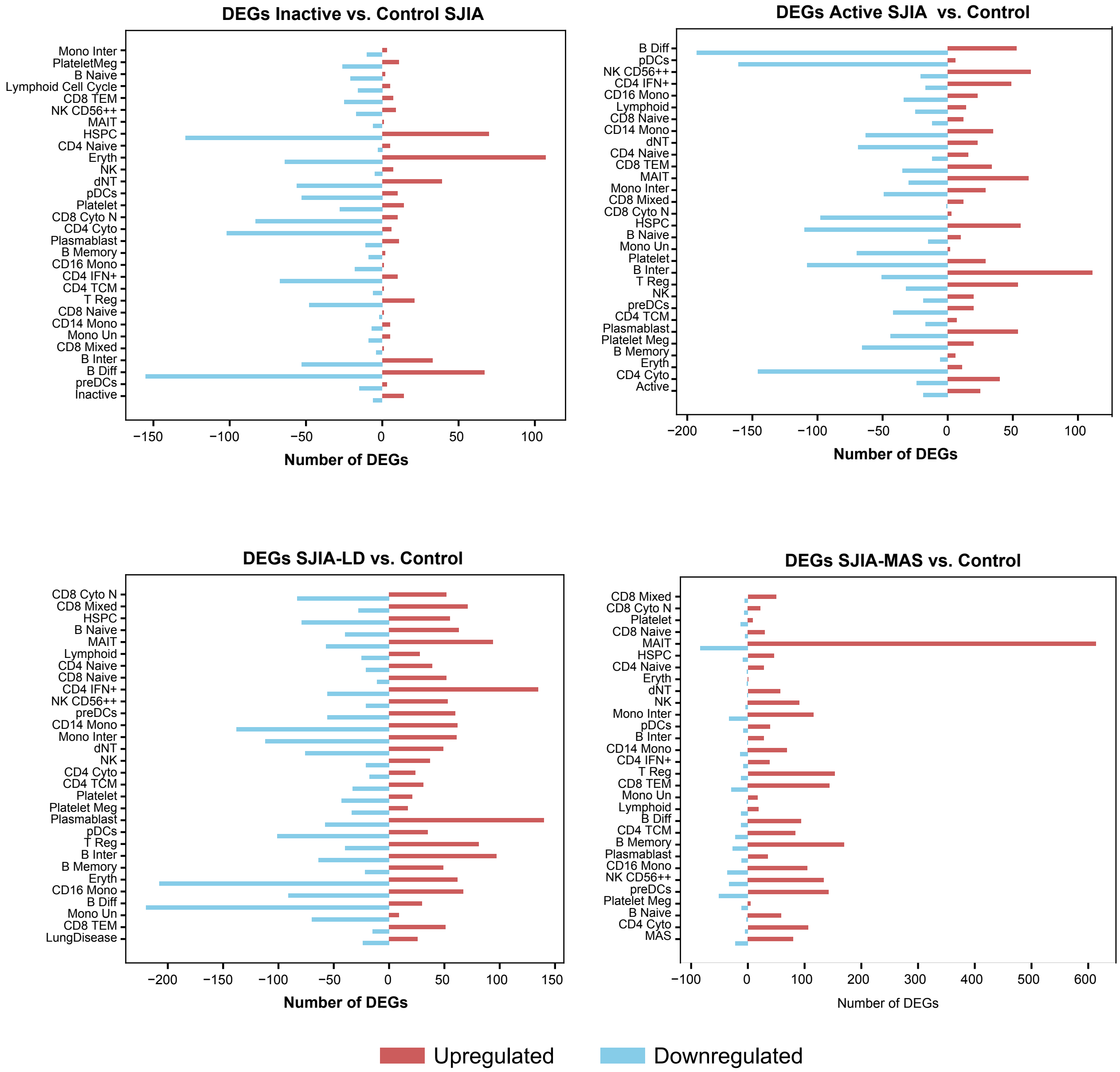

Supplementary Figure 3. Magnitude of cellular gene expression inpacts in each SJIA subtype. Gene summary for cellHarmony comparisons, showing overview of up- and downregulated genes for each cell type (cut-off for Contr vs MAS rawp ≤ 0.005, all other comparisons adj. p-value ≤ 0.05) .

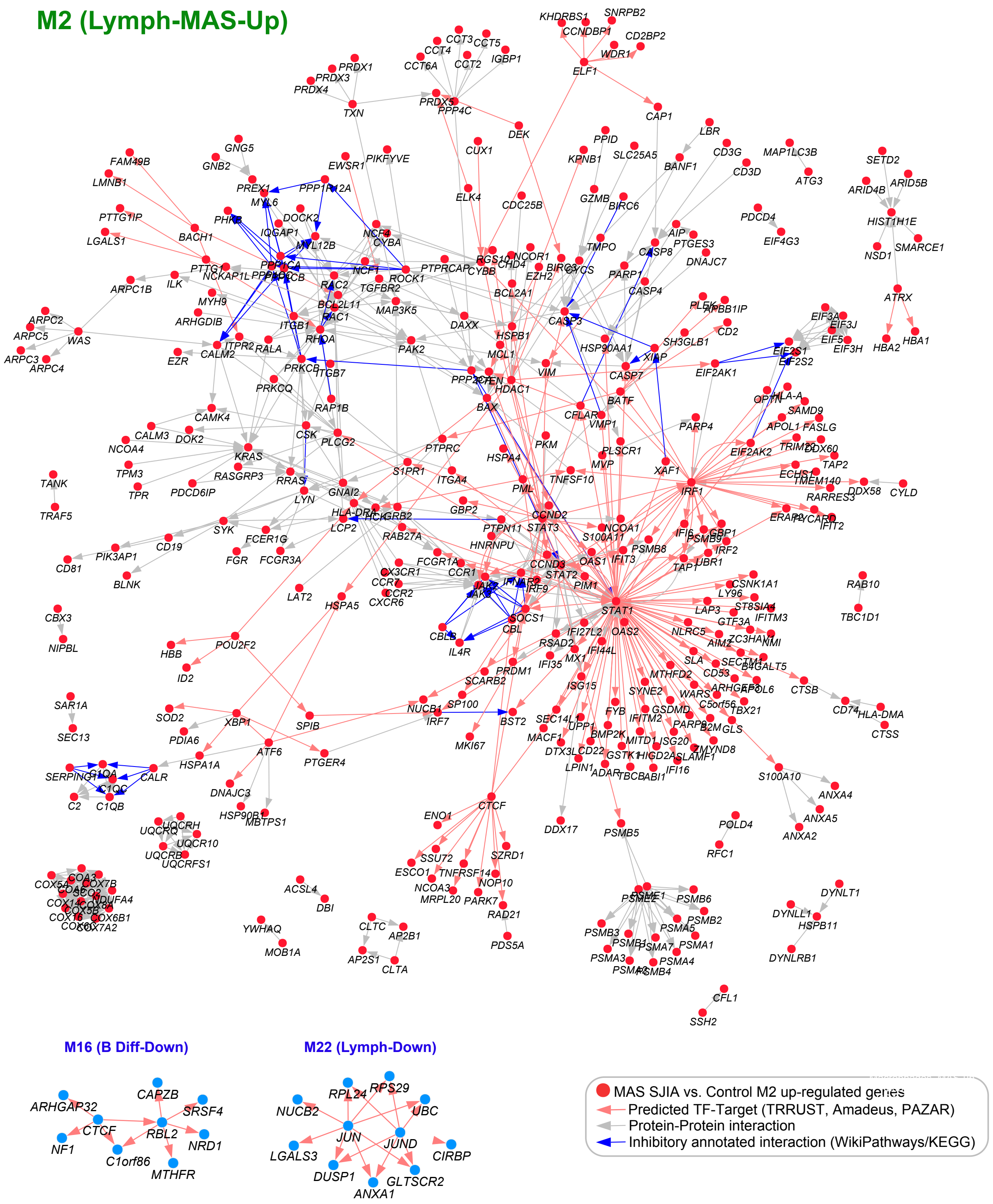

Supplementary Figure 4. Transcriptional networks underlying SJIA-MAS lymphoid gene regulation. Predicted reglulatory network (NetPerspective) for any common genes in SJIA exemplar populations. (Top) SJIA-MAS lymphoid populations (n=22 MAS vs. control comparisons) upregulated gene module (M2). (Bottom) SJIA down-regulated modules with complex SJIA clinical subtypes. Central nodes represent putative master regulatory control transcription factors upstream of the denoted targets.

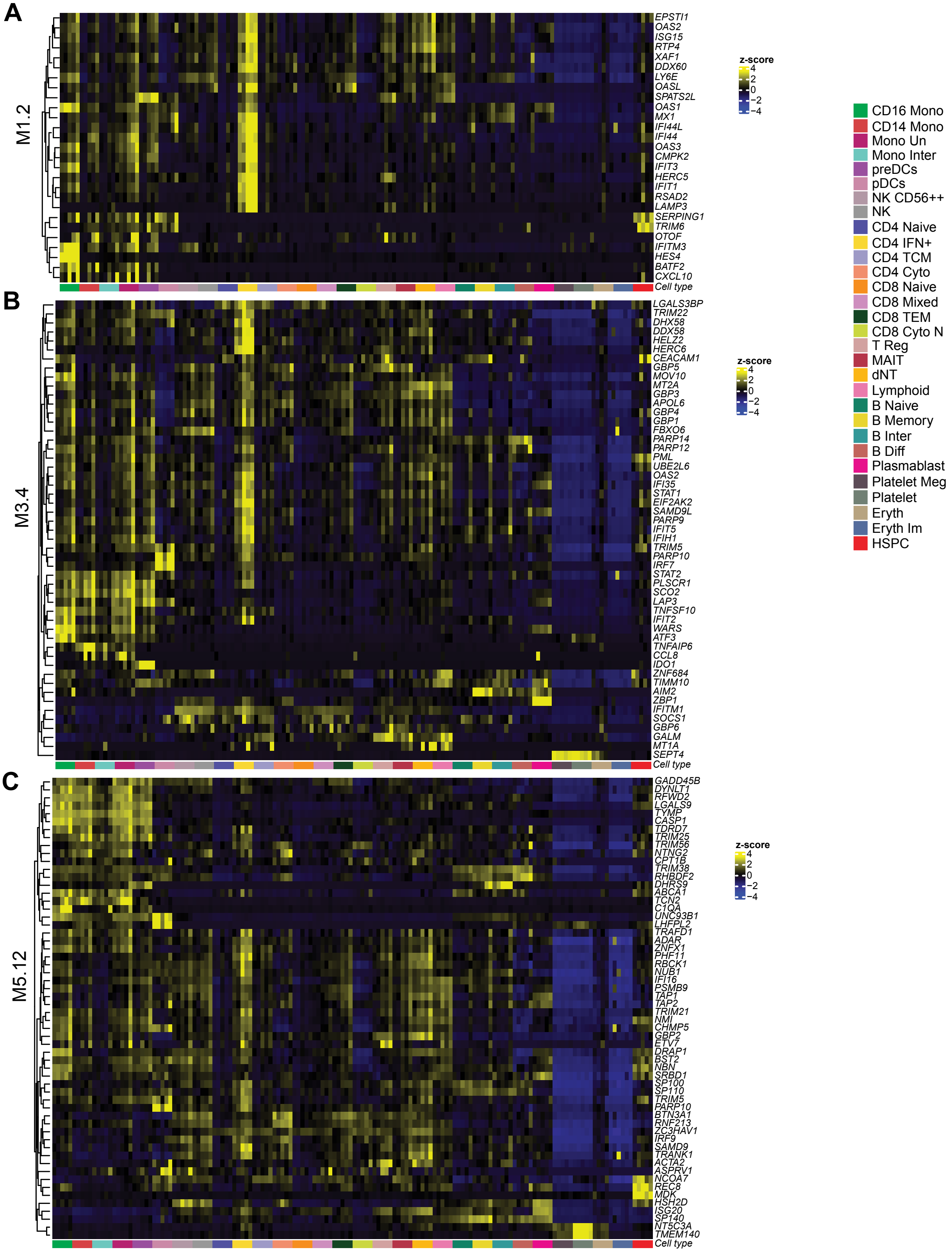

Supplementary Figure 5. Variation in the cellular source for different IFN mediated genes. A-C) Heatmaps of IFN induced genes organized by cell type. A) Module M1.2, B) Module M3.4, C) Module 5.12 as previously described in Banchereau et al., 2016.

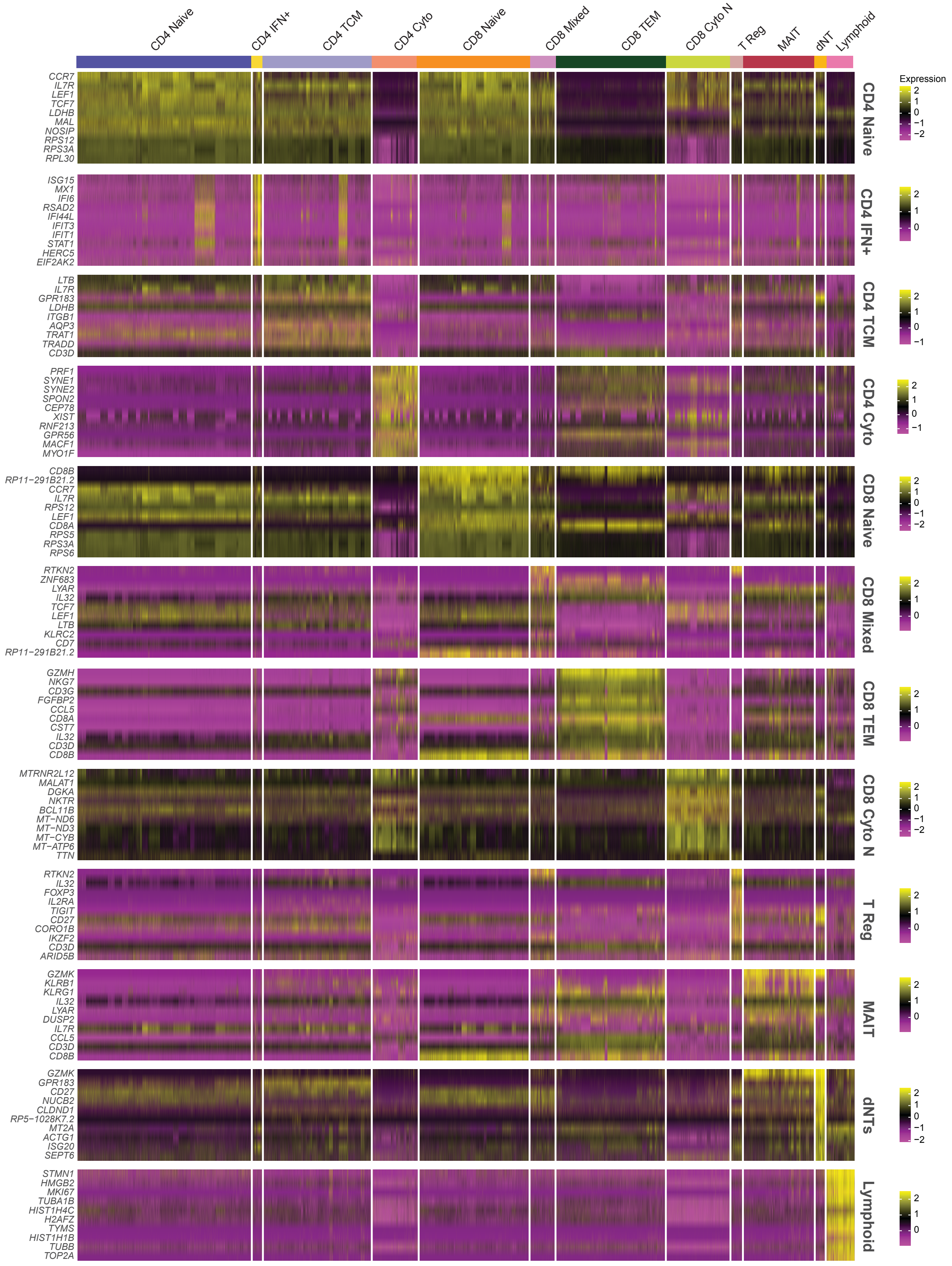

Supplementary Figure 6. T-cell population markers in all cells. Top 10 Seurat defined marker genes for each of the 12 T-cell annotated cell populations.

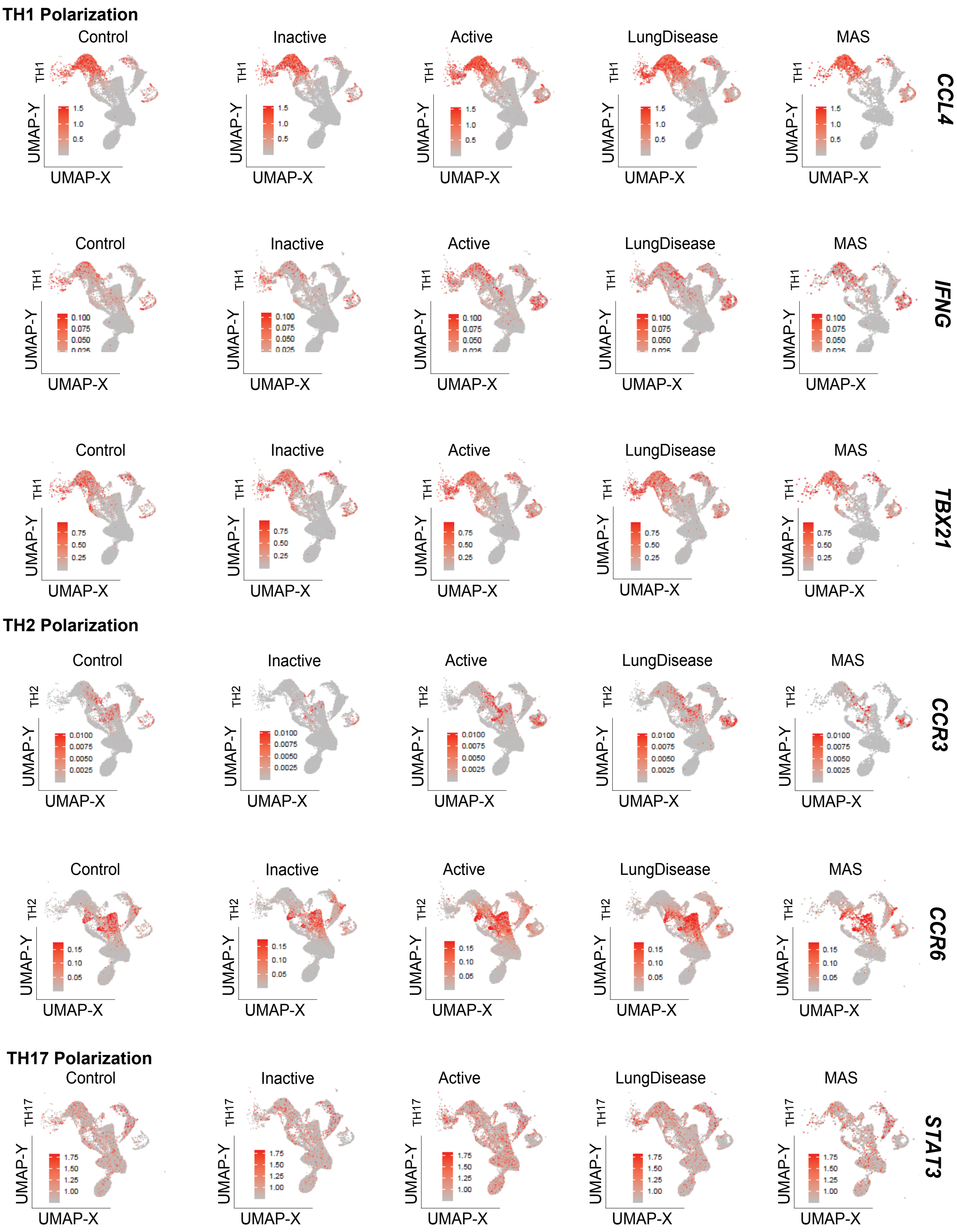

Supplementary Figure 7. T cell polarization markers expressed in the T cell populations. Expression of CCL4, IFNG and TBX21 gene expression indicating Th1 lineage, CCR3 or CCR6 as Th2 markers and STAT3 displaying Th17 polarization.

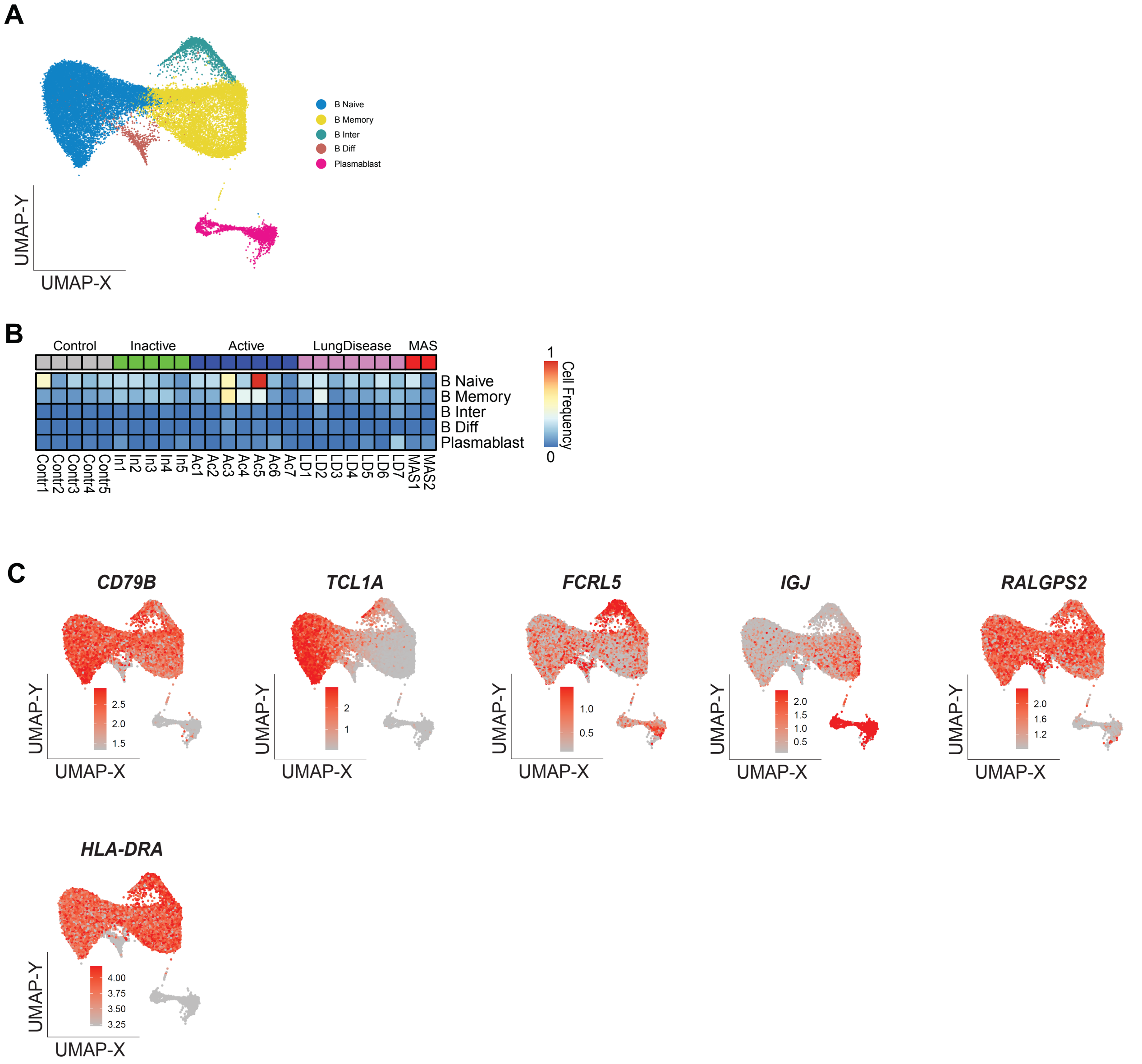

Supplementary Figure 8. Population specific marker gene expression in distinct B-cell subsets. A) UMAP representing 5 B cell populations identified by scRNA-seq. B) Matrix representing cell frequency per individual SJIA patient or control for 5 B cell populations. C) Feature plots representing expression levels of selected B cell population marker genes.

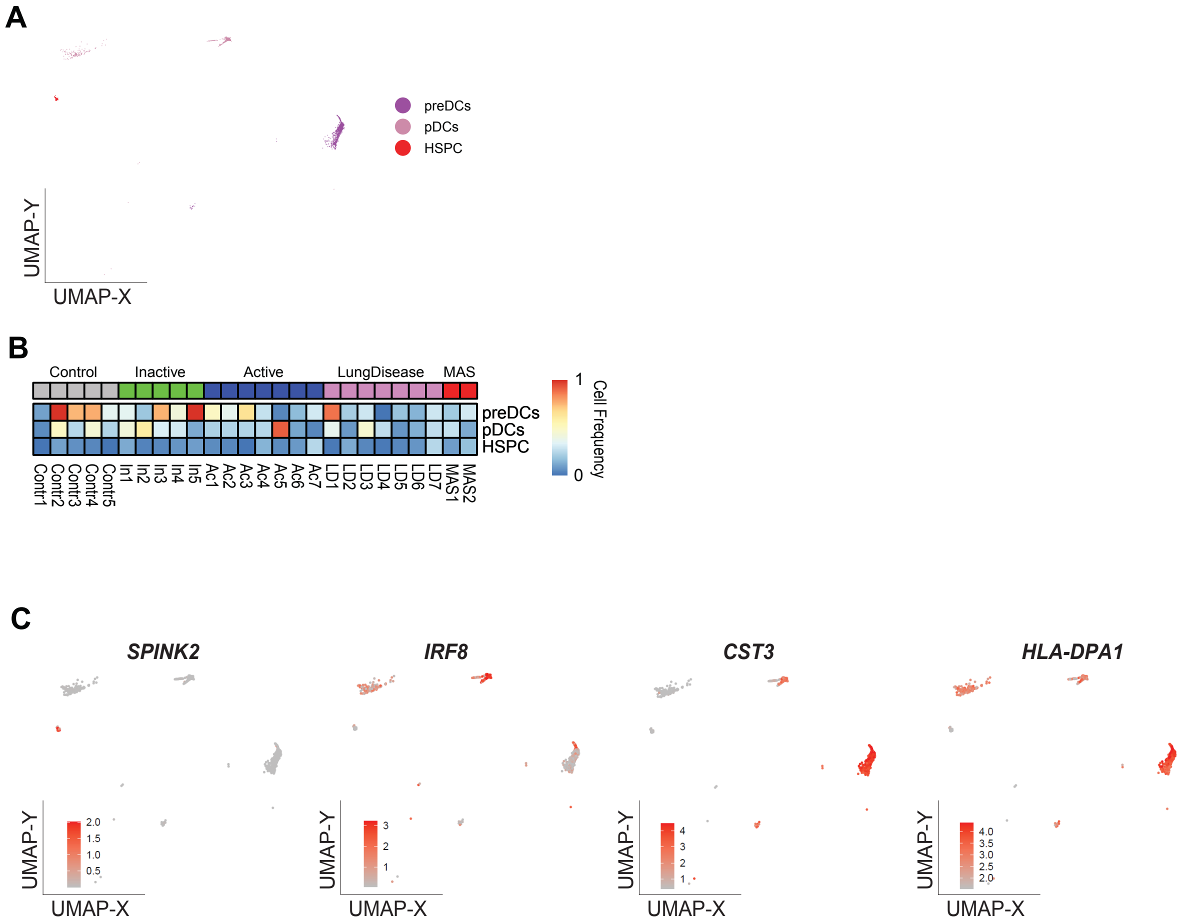

Supplementary Figure 9. Dendritic cell populations in SJIA. A) UMAP representing 2 DC cell populations and 1 HSPC population identified by scRNA-seq. B) Matrix representing cell frequency per individual SJIA patient or control for selected populations. C) Feature plots representing marker gene expression levels of 2 DC cell populations and 1 HSPC population.

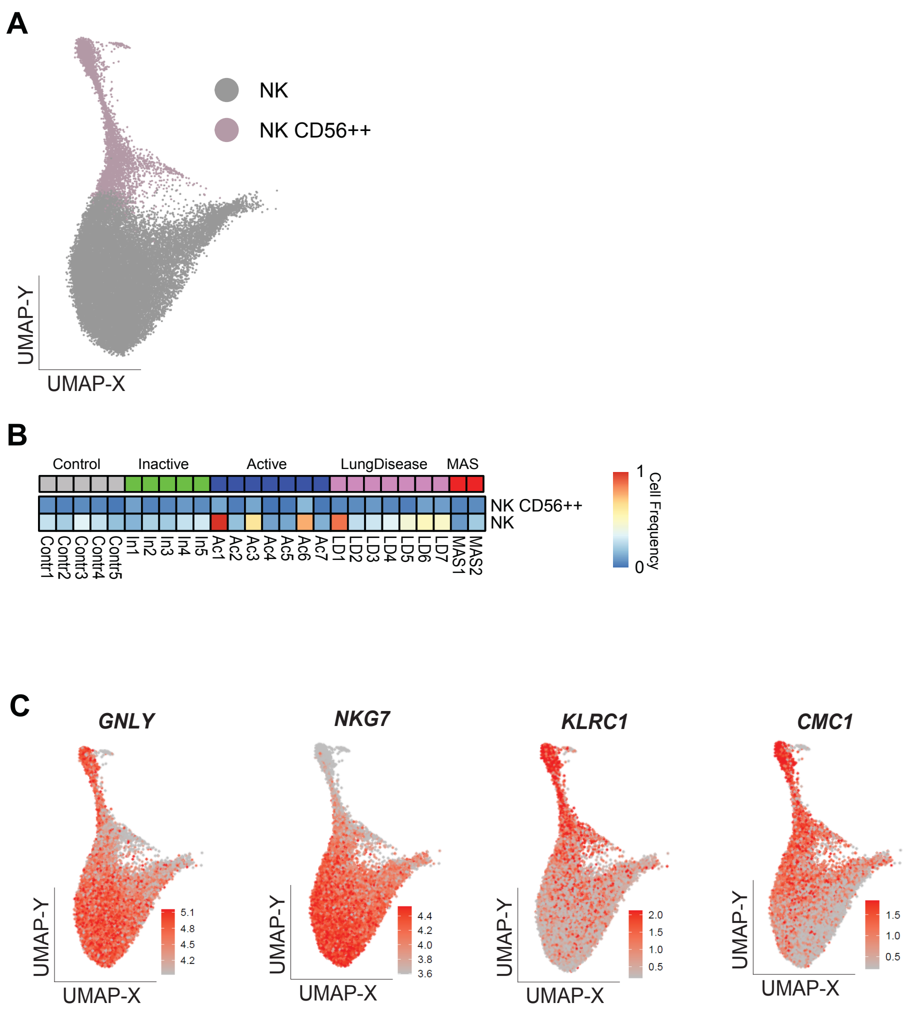

Supplementary Figure 10. NK cell populations in SJIA. A) UMAP representing 2 NK cell populations identified by scRNA-seq. B) Matrix representing cell frequency per individual SJIA patient or control for 2 NK cell populations. C) Feature plots representing expression levels of selected NK cell population marker genes.

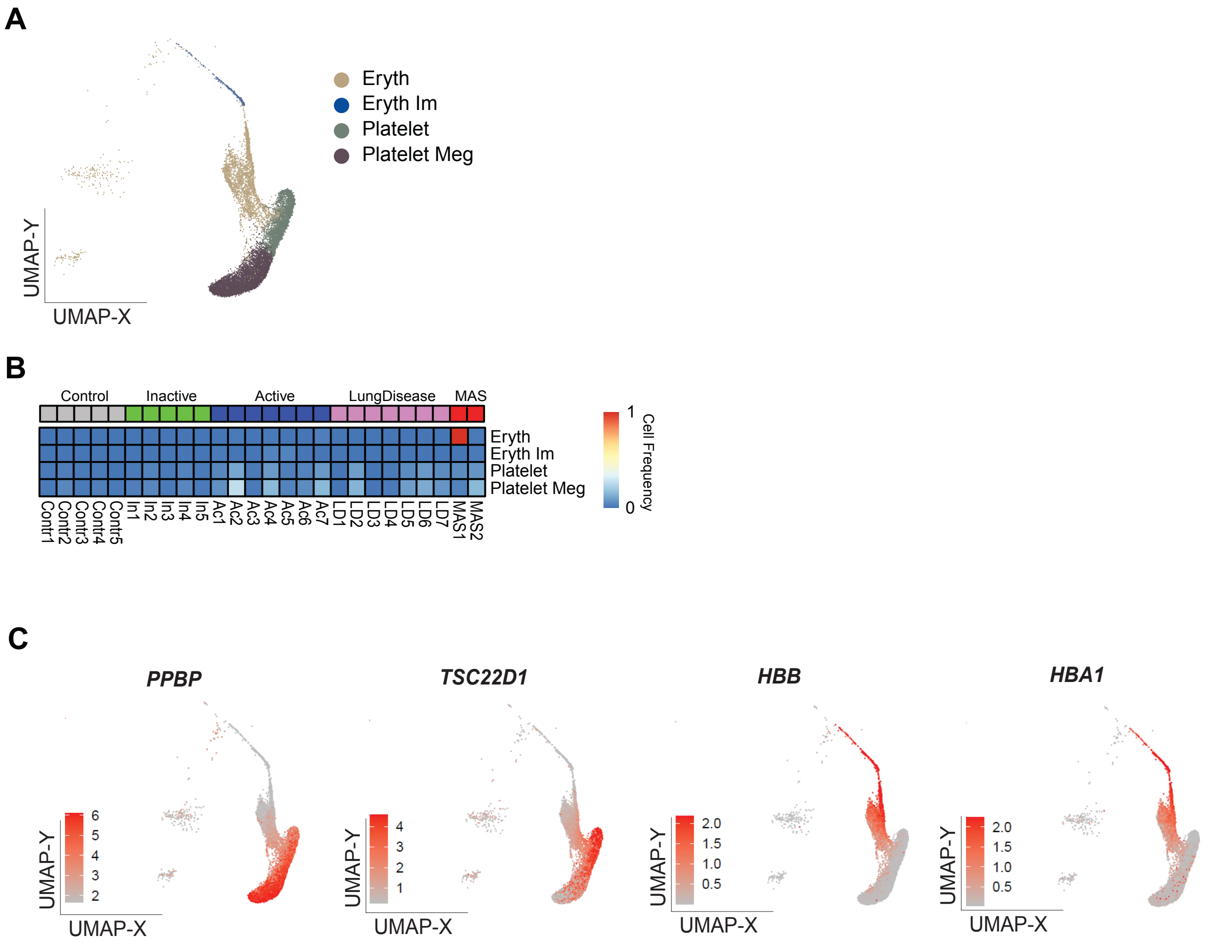

Supplementary Figure 11. Erythroid-megakaryocytic cell populations in SJIA. A) UMAP representing 2 Erythrocyte and 2 Platelet cell populations identified by scRNA-seq. B) Matrix representing cell frequency per individual SJIA patient or control for selected populations. C) Feature plots representing marker gene expression levels of Erythrocyte and Platelet marker genes.

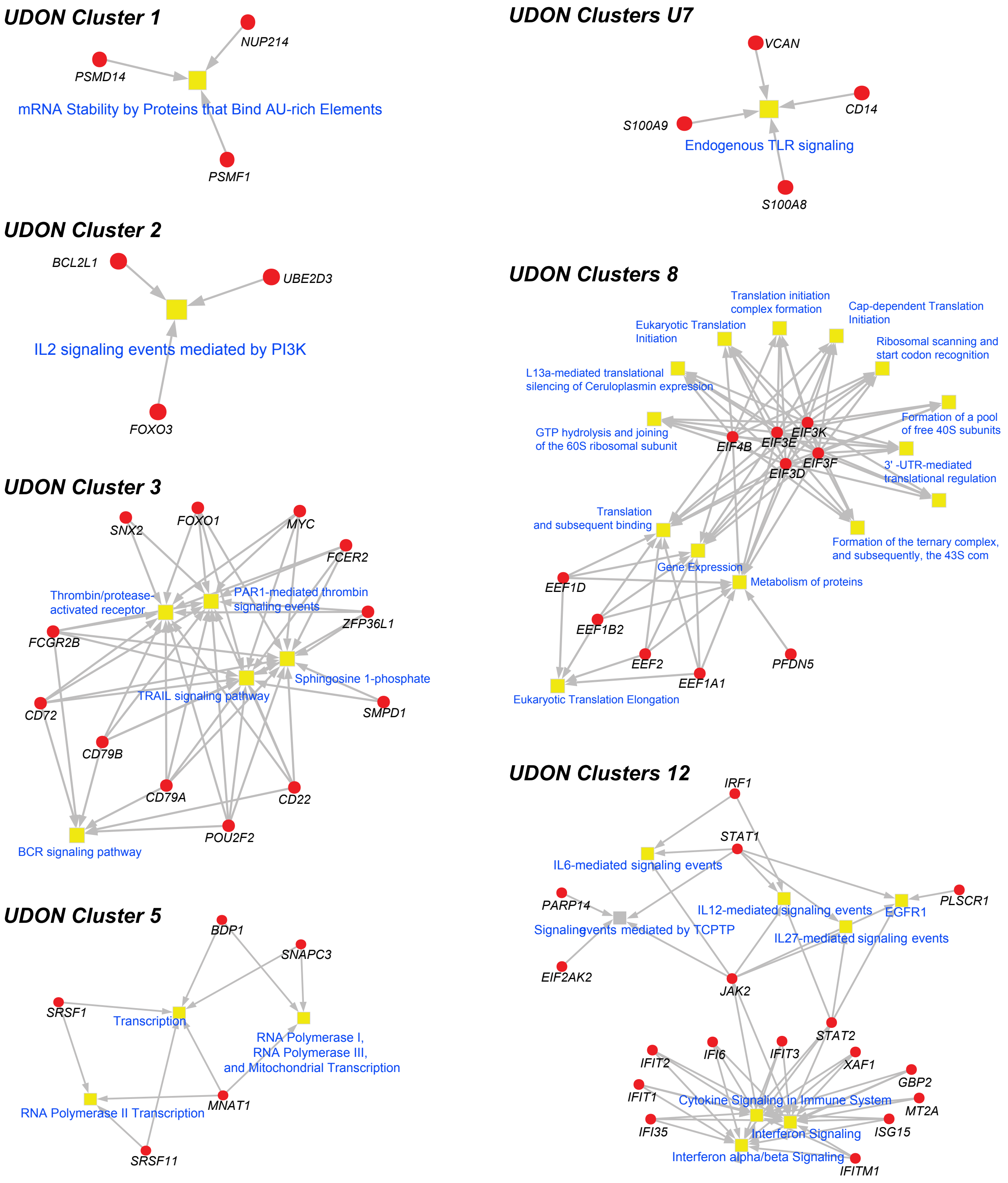

Supplementary Figure 12. UDON cluster enriched pathway genes. Genes and biological pathways (PathwayCommons) enriched in UDON analysis of SJIA patient pseudo-bulk fold clusters (GO-Elite).

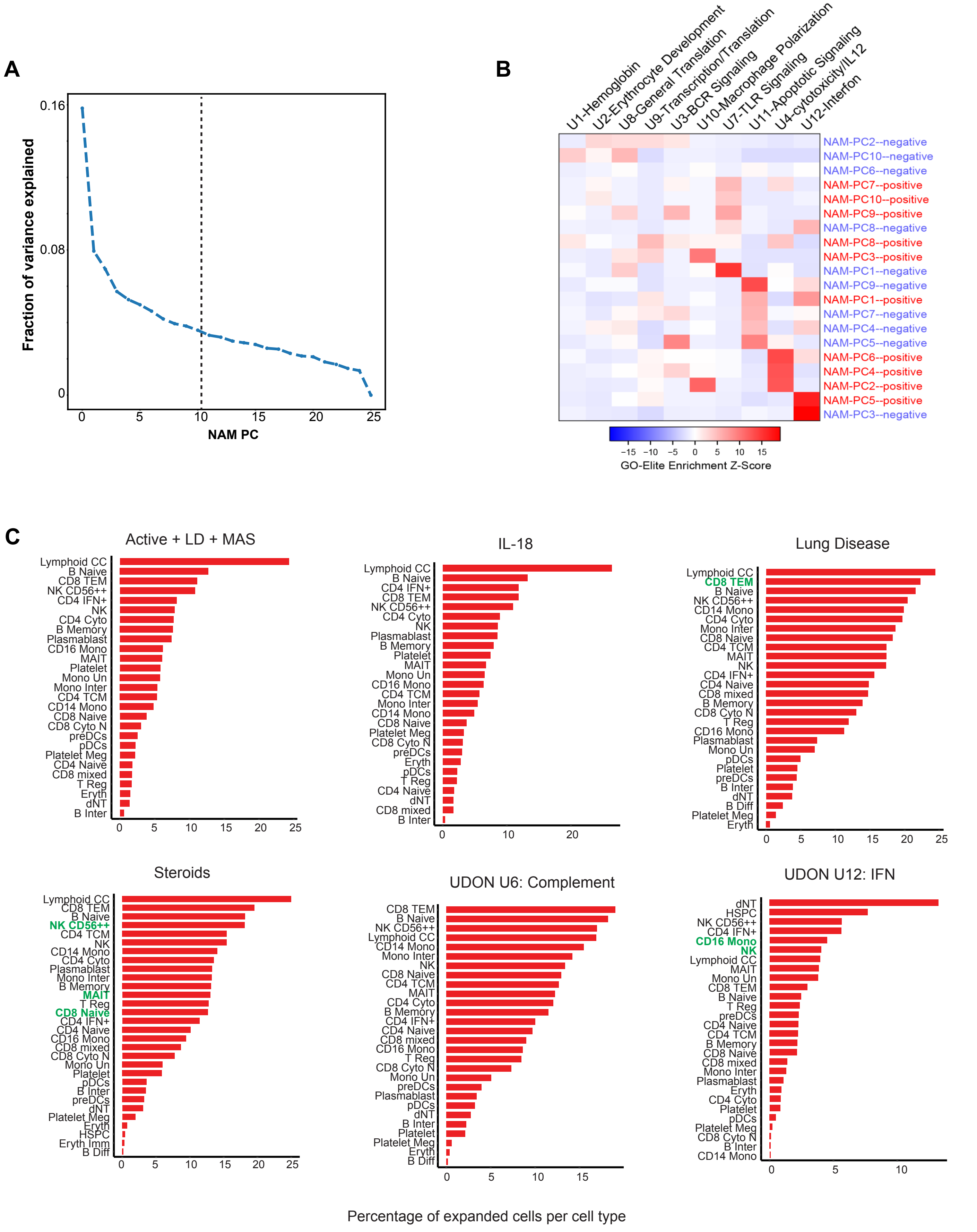

Supplementary Figure 13. Comparison of CNA NAM-PC gene sets to UDON cluster marker genes. A) Fraction of variance explained by each neighborhood adjacency matrix principal component (NAM PC) identified by CNA. B) Heatmap from the software GO-Elite, displaying gene-set enrichment z-scores for all queried NAM-PC positive and negative loading genes (n=100). C) Enrichment of cell types in CNA's expanded populations associated with clinical phenotypes and UDON subtypes in the SJIA patient cohort. Bar plot indicates the percentage of CNA expanded cells by the total number of cells per cell type. Associations also identified by SATAY-UDON are colored in green.

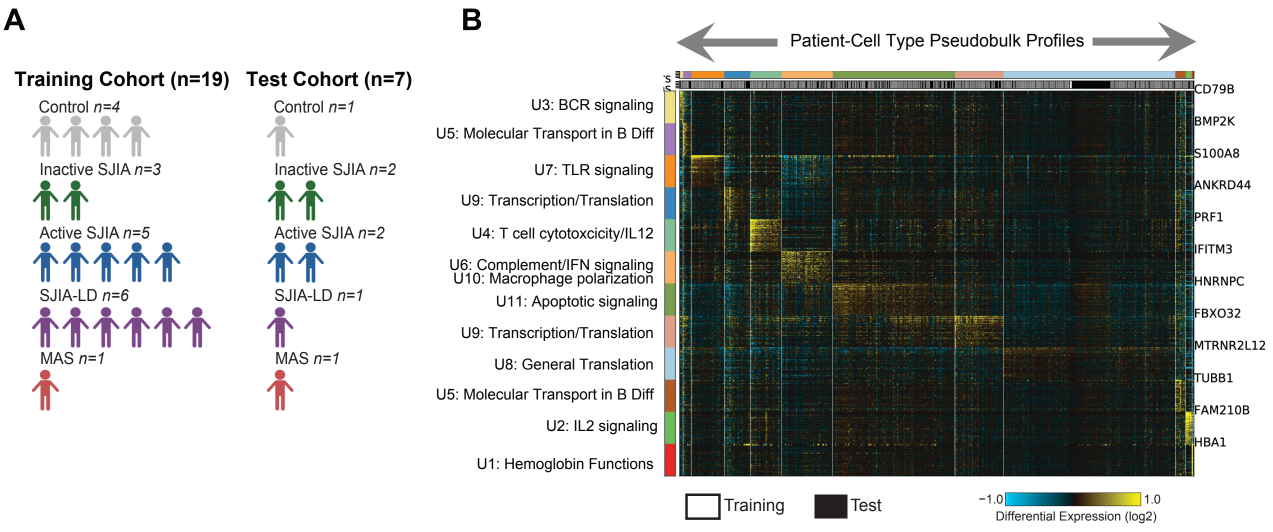

C

Clinical Phenotype	Inactive	Active	Lung Disease	MAS
IL-1 inhibition	☑ (green)		☑ (red)	
IL6R inhibition	☑ (red)			
Steroids			☑ (green)	☑ (blue)
Ferritin				
CXCL9			☑ (green)	☑ (green)
IL-18				
CRP		☑ (blue)		
S100A8/A9				
S100A12				
Systemic Features				
Active Arthritis		☑ (green)		
Fever				☑ (green)
ANC 1000X				

Supplementary Figure 14. UDON reproducibly finds novel SJIA patient subtypes. A) The SJIA patient cohort was split into 19 training and 7 test samples. B) Heatmap pseudobulk-fold heatmap of the excluded test samples reclassified into re-derived UDON clusters on the 19 sample training dataset. C) UDON-SATAY enriched analysis results comparing associations specifically idenfitied in the training dataset association (blue check mark), those detected in both the training and original (all samples) UDON analysis (green check mark) and those only detected in the original UDON analysis (red check mark).

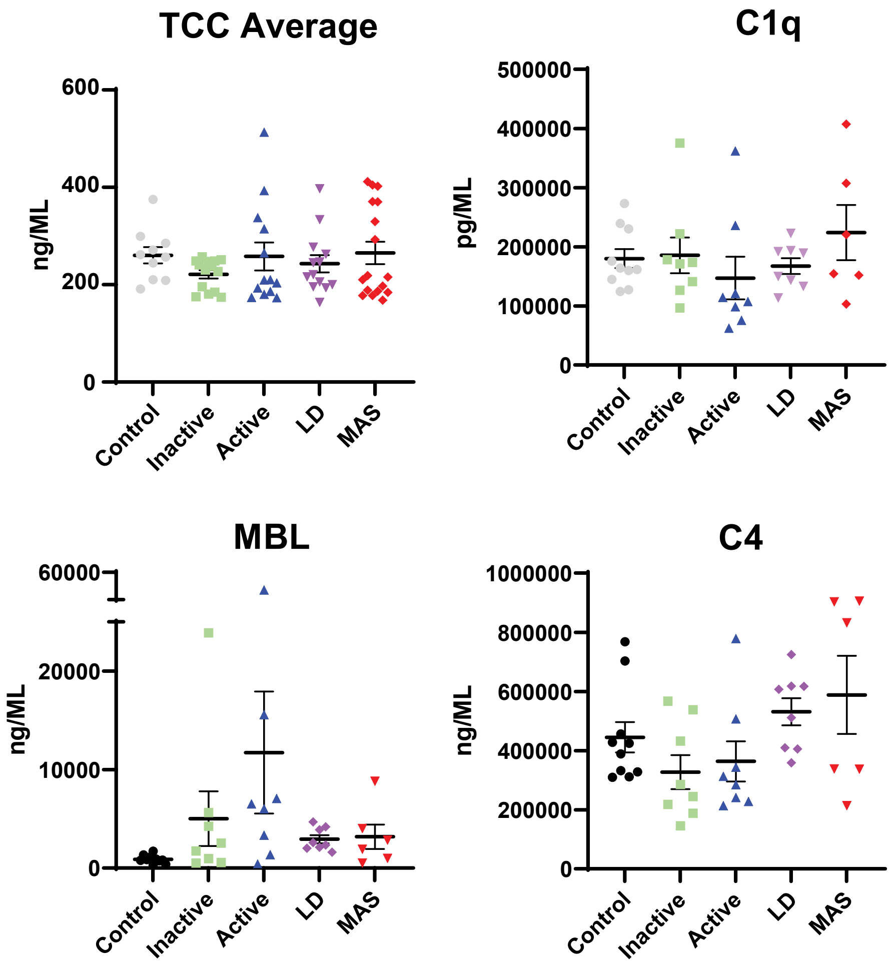

Supplementary Figure 15. Complement activation pathway serum protein evaluation in SJIA. TCC Average, C1q, MBL (Mannose binding lectin) and C4 levels were measured by LUMINEX. Significant differences were calculated by one-way ANOVA and depicted as *= adj. p-value ≤ 0.05.

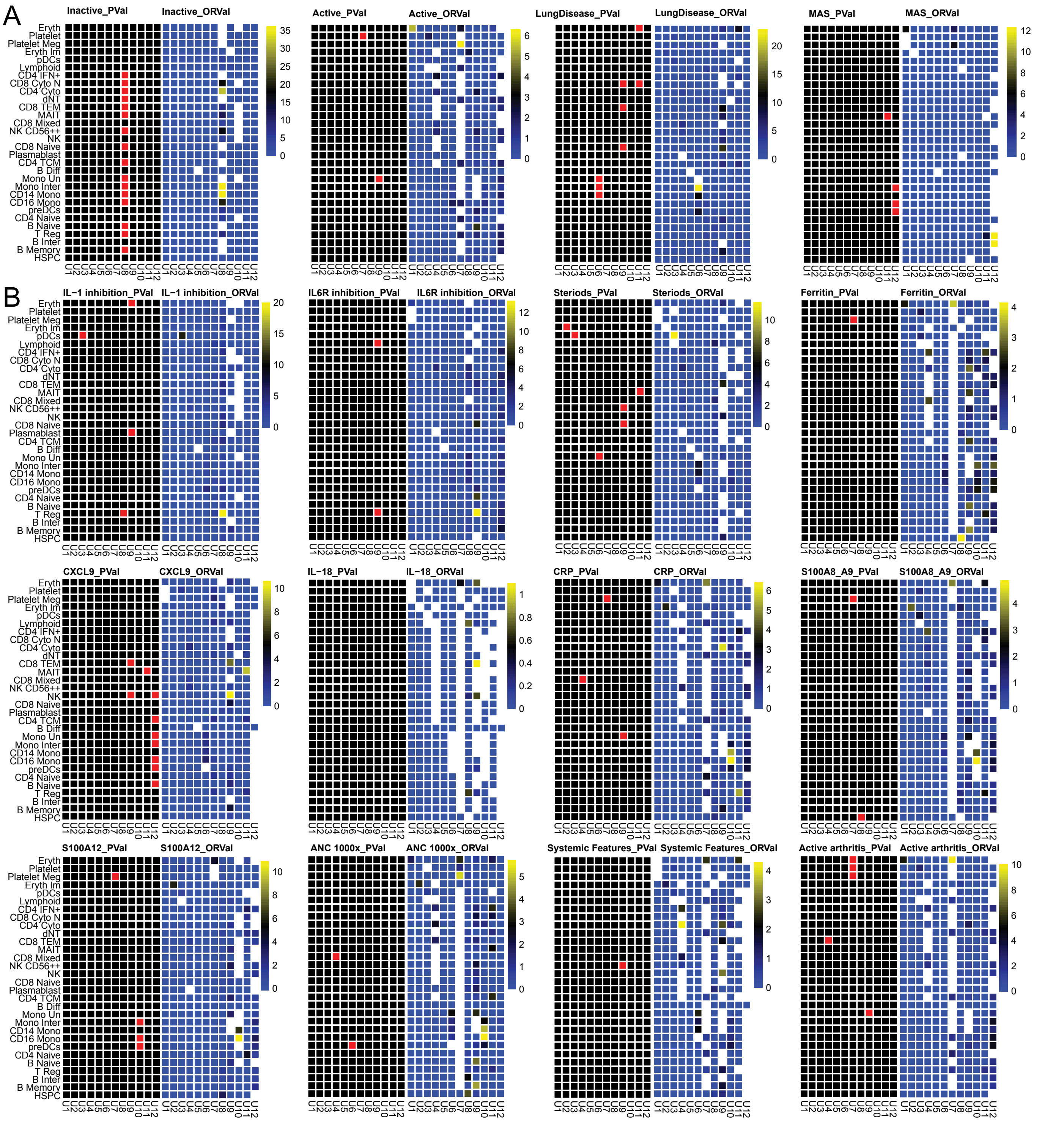

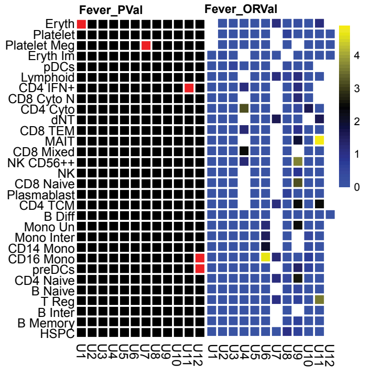

Supplementary Figure 16. A) Odds Ratio values (ORVal) for UDON SATAY results of patient group/cell type associations. B) UDON SATAY results for each clinical parameter or treatment, displaying significant associations by p-value (red box = p<0.05) and Odds Ratio values per cell type.

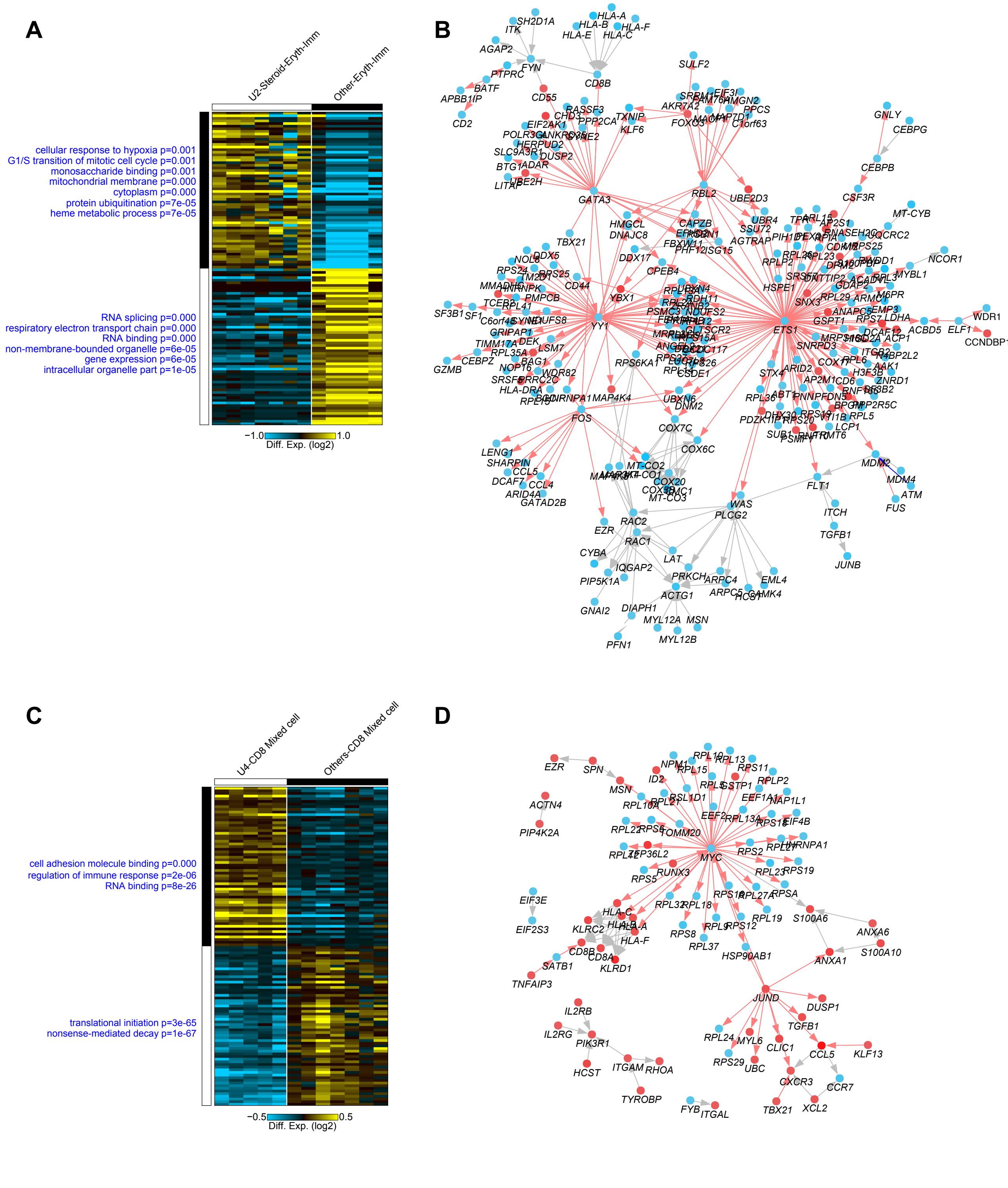

Supplementary Figure 17. Transcriptional analysis of SATAY-UDON associations. A) MarkerFinder analysis and B) DEG network (p≤0.05) comparing gene programs of Eryth-Immature Pseudobulks of U2 vs. Eryth-Immature in other UDON Clusters. C) MarkerFinder analysis and D) DEG network (p≤0.05) comparing gene programs of CD8 Mixed Pseudobulks of U4 vs. CD8 Mixed in other UDON Clusters. Enrichment of PathwayCommons gene sets from GO-Elite are indicated on the left of the heatmaps in panels A and C.

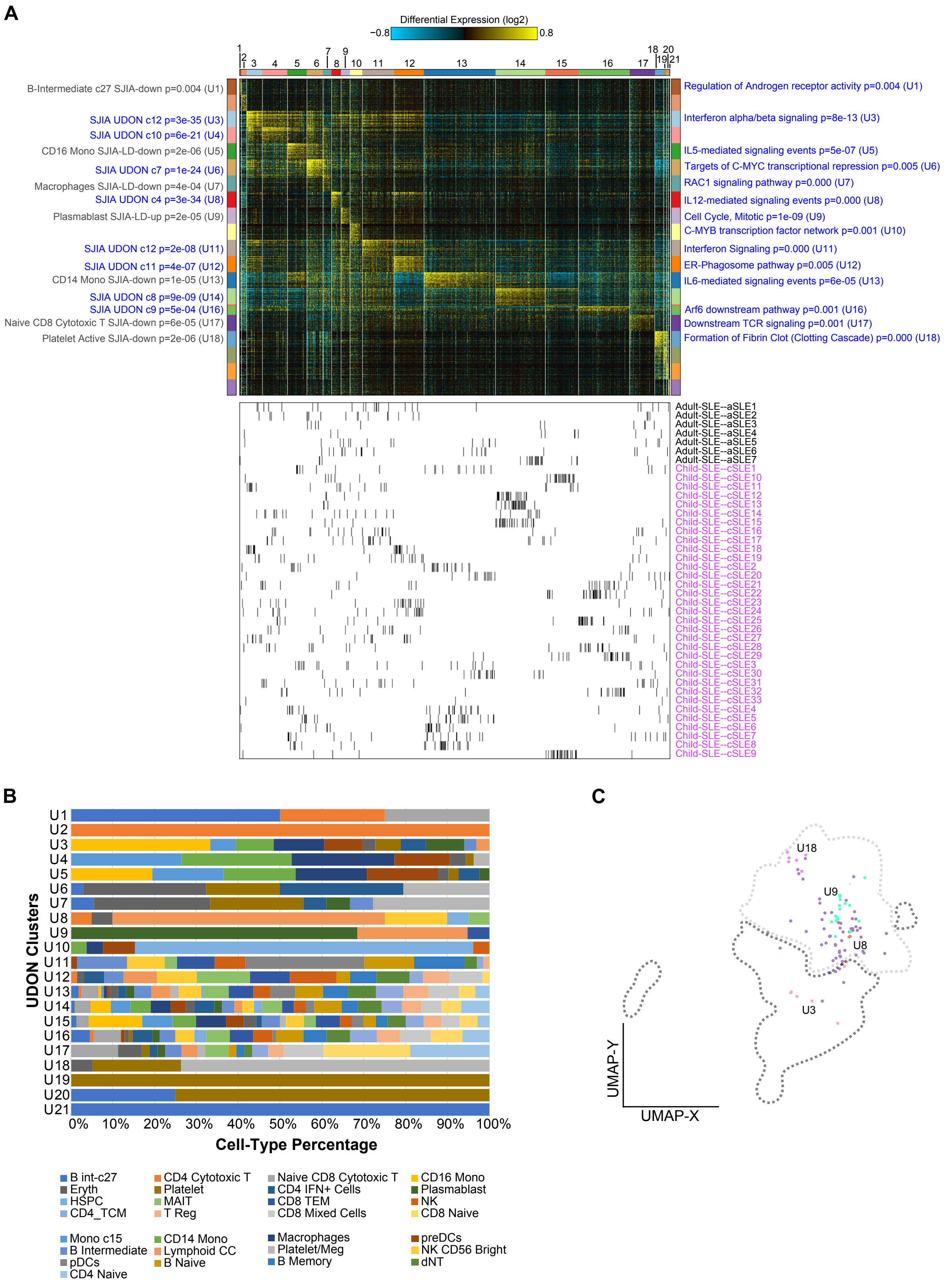

Supplementary Figure 18. UDON analysis of child and adult SLE. A) Heatmap of SLE-UDON clusters. Columns = patient pseudobulk folds, Rows=marker genes. Enrichment of SJIA (left) and PathwayCommons (right) gene sets from GO-Elite. Matrix of pseudobulks by donor are displayed below the heatmap. B) Matrix representing cell frequency per individual UDON cluster. C) Projection of SLE-UDON cluster labels onto OneK1K pseudobulk folds.

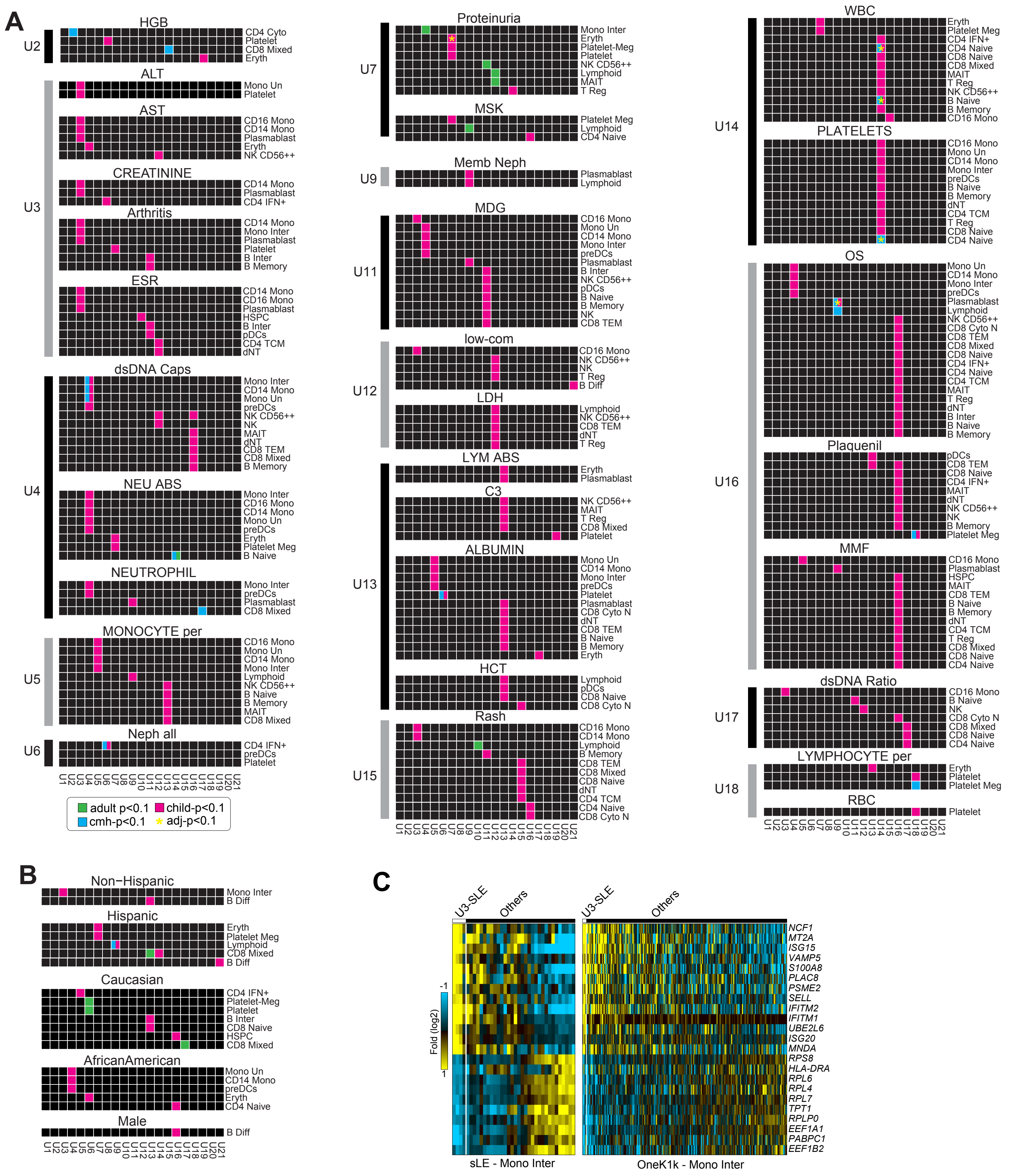

Supplementary Figure 19. UDON identifies novel clinical biomarker associations in SLE. Age-independent and age-specific SATAY UDON results for A) each SLE clinical parameter , or B) race and gender of SLE patient, displaying significant associations by p-value (blue = p<0.10 = Cochran-Mantel-Haenszel test, green = p<0.1 adult-specific one-sided Fisher's Exact test, pink = p < 0.1 child-specific one-sided Fisher's Exact Test, yellow star = FDR-adjusted p < 0.1). C) Heatmap of common differentially expressed genes (fold>1.2 and empirical Bayes t-test p<0.05, two sided), for SLE UDON cluster 3 intermediate moncyte pseudobulk folds versus other intermediate moncyte in the SLE and OneK1K cohort (OneK1K Others subsampled for visualization).

Single-cell RNA-Seq Analysis

Read alignment and Cell Clustering. Raw FASTQ files were aligned to hg19 reference genome and transcriptome and provided as input for CellRanger version 3 to generate counts matrices for all samples. Seurat integrated analysis was performed on count matrices from all donor samples using Seurat version 3. After visualizing the number of unique reads and the percentage of mitochondrial counts in each cell, we first removed cells with unique feature counts over 6,000 or less than 100 and cells that have more than 25% mitochondrial counts to remove low-quality cells. We applied the default VST method to identify highly variable genes from each sample and CCA on the first 50 CCA dimensions to integrate the samples. Here, we applied author recommendations to scale the integrated data and performed principal component analysis (PCA) on first 50 dimensions. UMAP was derived from the first 36 PCs, determined by the elbow plot method. We chose 30 clusters (cluster resolution = 0.6), which resulted in clusters with unique marker gene expression (see Results). The marker genes of each cluster were determined using the *FindAllMarkers* function. Unique marker genes for each cluster were selected based on maximum average log fold change value for a gene for a given cluster (**Supplementary Table 4**). Clusters annotations were guided by Azimuth PBMC assignments and refined based on a literature search for enriched marker genes (56).

Differential Gene Expression Analyses and Supervised Clustering. The software cellHarmony was used to perform pairwise differential expression comparisons between patient cell-population pseudobulks. For this analysis, pseudobulks were computed in AltAnalyze using the sampleIndexSelection module with centroid=True. A cellHarmony labels file was created to guide the comparisons, indicating the pseudobulk clinical subtype (e.g., SJIA-MAS), query and reference groups. cellHarmony was then run using the --referenceType None (use provided clusters rather than derive through label transfer) and fold >1.2 and empirical Bayes moderated *t*-test $P < 0.005$, unadjusted. We compared the pseudobulks of active, lung disease, and MAS patients against the control patients, or combination of SJIA active, lung and MAS to controls. cellHarmony produced 153 differentially expressed gene-sets (DEGs) corresponding to all cell-type specific and broad (e.g., MAS vs. controls) comparisons. The DEG sets were separated into up- and down-regulated lists and combined to form a custom gene-set reference databases (text file) for the gene set enrichment software GO-Elite (57). These sample individual DEG sets (n=306) were provided as input to GO-Elite within AltAnalyze for comparison with this custom reference to derive all possible pairwise comparison overlaps and enrichments. The resulting pairwise z-score gene-set enrichments from GO-Elite were clustered in AltAnalyze (HOPACH

cosine clustering) to derive the pairwise enrichment matrix to determine shared DEG modules across different SJIA clinical subtypes and cell-populations. Modules were defined as groups of 3 or more terms with mutual relative enrichment (visually determined from the pairwise heatmap matrix). Module genes were defined as those which where common to at least two distinct SJIA comparisons in the annotated module, prior to reanalysis in GO-Elite (Gene Ontology enrichment). The algorithm MarkerFinder in AltAnalyze was used to rank enriched GO terms with most specific pattern to that module for display (58).

Covarying Neighborhood Analysis (CNA) in SJIA

To apply CNA to the SJIA cohort, we first downsampled the dataset from 209,955 to 54,264 cells by randomly selecting 100 cells from each cell cluster for each donor. To enable equivalent comparison of UDON clusters and CNA's neighborhood abundance matrix principal components (NAM-PCs), we applied CNA without covariate association testing. NAM was computed using the cna.tl.nam function, from the counts matrix of all samples and experimentally performed batches. CNA determines the neighborhood loadings for the first 10 NAM-PCs. For association-independent analysis, we consider the neighborhood loadings. Gene set enrichment (GO-Elite) was performed on the top-100 positive and top-100 negative correlated genes for the neighborhood loadings for the top 10 NAM-PCs, against the top 200 marker genes from each UDON cluster (MarkerFinder defined) (**Supplementary Table 7**). For this analysis, UDON cluster matches were determined when a gene-set overlap of >20% of the UDON cluster marker genes with the NAM-PCs positive or negative markers was obtained, in addition to a GO-Elite Fisher Exact $p < 0.05$ (FDR corrected). To find disease-specific associations using CNA, we applied cna.tl.association function to calculate the neighborhood coefficient. Specifically, for our SJIA dataset, we defined the cells that are from only the patient samples that are in the active, lung or MAS phase of disease as the "Disease" phenotype. This analysis excludes cells from the inactive samples in the disease cells. We applied the cna.tl.association function on the gene expression matrix with the "y" parameter set to the defined donors/samples, accounting for batch effects (author recommended workflow). We visualized the neighborhood coefficient, calculated by CNA, of cells that passed the FDR corrected p-value < 0.05. For each cell type in the dataset, we calculated the percentage of cells that have a positive neighborhood coefficient, also referred to as "expanded populations", by the total number of cells in the cell type.

We repeated the above for each individual SJIA disease subtype as well as 13 clinical phenotypes (e.g., IL18, fever). We also repeated the above for patients associated with specific UDON clusters. This was done by setting the phenotype of the UDON subtype-associated cells to the

specific UDON subtype (for example, setting the phenotype of CD16 Monocytes of patients A, B, and C to U12 if the UDON cluster (U12) comprised of CD16 monocytes pseudobulks from patients A, B, and C). We provide the input data files in Synapse (https://www.synapse.org/#!Synapse:syn52160307) and the CNA functional calls applied here in GitHub (https://github.com/kairaveet/udon-sjia-sle/tree/main/cna_evals).

External Dataset Analyses

scRNA-Seq datasets. Author distributed count matrices (CellRanger derived outputs) were directly obtained from the Gene Expression Omnibus database for SLE (GSE135779) (2) and OneK1K (GSE196735) (3) cohorts. These data were further processed and aggregated using the software AltAnalyze version 2.1.4. Count matrices were scaled to Counts per ten thousand reads per gene (CPTT), merged and aligned to the 30 PBMC cell population centroids (healthy controls only) using cellHarmony with default centroid alignment options. Pseudobulk folds were computed for aSLE and cSLE against their matching controls using the sampleIndexSelection module of AltAnalyze with the parameters: --centroid True, --fold True and –removeNegatives True. Conversely, for the OneK1K cohort, pseudobulks were computed for all samples and cell-types, with the mean of all pseudobulks considered as a reference for fold calculation using the above mentioned sampleIndexSelection script. For SLE analysis, we applied UDON on the resulting pseudobulk folds as described above. SLE pseudobulk folds were aligned to SJIA UDON cluster centroids using cellHarmony, whereas OneK1K pseudobulk folds were aligned to both SJIA and SLE UDON cluster centroids. For visualization, pseudobulk folds from all analyzed samples were jointly projected into a common UMAP embedding using python library scikit-learn to train and transform the UDON markers using PCA (top 50 components).

Bulk transcriptomics datasets. For independent SJIA bulk transcriptomics validation, pre-processed and normalized SJIA microarray gene expression data were downloaded from GEO (GSE80060, GSE7753) and median normalized (log2 expression). The genes in this dataset were restricted to those from the UDON clusters and ordered accordingly.
